# A co-evolved peptide-GPCR system senses host entry to drive fungal infection

**DOI:** 10.1038/s41467-026-78428-6

**Published:** 2026-10-09

**Authors:** Gabriel Mendoza-Rojas, Philip Nakonz, Min Lu, Nora Marie Kühne, Johannes Postma, Naomi Shtakser, Max Heinen, Manav Patel, Orlando Arguello-Miranda, Kai Heimel, Sonja Billerbeck, Florian Altegoer

**Affiliations:** 1 Heinrich-Heine University Düsseldorf, Institute of Microbiology, Cluster of Excellence on Plant Sciences, Düsseldorf, Germany; 2 University of Groningen, Faculty of Science and Engineering, Groningen, Netherlands; 3 Georg-August University Göttingen, Microbial Cell Biology, Göttingen, Germany; 4 NC State University, Plant and Microbial Biology, Raleigh, USA; 5 Imperial College London, Department of Bioengineering, London, UK

**Keywords:** Fungal pathogenesis, Fungal biology, G protein-coupled receptors, Pathogens

## Abstract

A successful infection requires pathogens to recognize the specific host environment in order to reprogram their physiology accordingly. One major way in which eukaryotic cells sense their surroundings is via G-Protein Coupled Receptors (GPCRs), which share a seven-transmembrane architecture and G-protein-mediated downstream signaling. While mammalian GPCRs are well-characterized and represent important drug targets, their fungal counterparts remain poorly understood. Here we uncover a GPCR-based mechanism that allows the corn pathogen *Ustilago maydis* to sense whether it has entered plant tissue. During infection, the fungus secretes the protein Pit2, which is cleaved by host apoplastic cysteine proteases, releasing a peptide ligand ‘hidden’ within the protein core. This ligand activates the fungal GPCR Gpe1, thus promoting fungal proliferation after initial host penetration. We elucidate the crystal structure of Pit2 and model the complex formed by Gpe1 and the Pit2-derived peptide. Structure-guided mutational analysis supports that disrupting this interaction reduces fungal virulence. The Gpe1/Pit2 system is conserved in related fungal species, with co-evolutionary signatures apparently preserving receptor-ligand specificity. Furthermore, this system shows mechanistic similarities to mammalian receptors, suggesting an evolutionary link between fungal and mammalian GPCRs.

## Abstract (web summary)

Eukaryotic cells can sense their surroundings via G-Protein Coupled Receptors (GPCRs). Here, Mendoza-Rojas et al. show that the plant pathogenic fungus *Ustilago maydis* perceives whether it has entered plant tissue by using a GPCR that is activated by a peptide released by host proteases upon cleavage of a secreted fungal protein.

## Introduction

Interactions between pathogenic fungi and their hosts depend on the pathogens ability to perceive and respond to host-derived signals that facilitate infection. One well-characterized way of eukaryotic cells to sense the environment occurs via G-protein coupled receptors (GPCRs), which share a seven-transmembrane architecture and conserved intracellular signaling pathways. The human genome encodes over 800 GPCRs, which together represent targets of nearly 40% of approved drugs, highlighting their central roles in physiology and disease^1^. Likewise, fungal genomes harbor numerous GPCRs, and emerging evidence indicates they contribute to colonization of humans and plants, although the repertoire of ligands and activation mechanisms remains poorly understood^2–5^. The smut fungus *Ustilago maydis* is a pathogen of corn and a model organism for studying plant-pathogen interactions and fungal development. Like other smut fungi, *U. maydis* undergoes a complex life cycle that includes a yeast-like stage and infectious hypha^6^. Hyphal development is tightly coordinated with the detection of host-derived cues^7^, which triggers the expression of virulence-associated effector genes. Although deletion of many effector genes results in only minor effects on pathogenicity^8,9^, a subset is essential, with loss-of-function mutants arresting infection prior to penetration^10^ or shortly thereafter^11^. These well-characterized examples include the effector Pit2, which is processed by host papain-like cysteine proteases (PLCPs) to release a peptide that inhibits PLCP activity^12,13^. Notably, *pit2* is genomically linked to *pit1*, a gene encoding a predicted transmembrane protein of unknown function whose deletion phenocopies the *pit2* deletion mutant^11^. Despite the extensive knowledge on effector gene functions, only little is known about how the fungus perceives the host, allowing to establish a successful infection. Although there is strong evidence for the importance of GPCR-mediated signaling^6,14–17^, canonical GPCRs have not yet been investigated in *U.maydis*, leaving a significant gap in our understanding of perception mechanisms in this pathogen.

In this work, we show that Pit1 encodes a fungal G-protein-coupled receptor, which we rename G-protein-coupled receptor promoting fungal *in planta* expansion 1 (Gpe1). This receptor is activated by a peptide released from Pit2 through processing by host papain-like cysteine proteases. Structural analysis of Pit2 reveals that the activating peptide is buried within the Pit2 homodimer, suggesting that Pit2 acts as a scaffold which prevents premature receptor activation. Structure-guided mutational analyses identify residues in both the peptide and Gpe1 that are required for receptor activation and fungal virulence. We further show that Gpe1 activation induces receptor internalization and promotes fungal proliferation, accompanied by increased expression of the cell-cycle regulator Clp1. Comparative analyses indicate that Gpe1 and Pit2 have co-evolved in smut fungi and that Gpe1 contains a conserved TM3–TM6 module with similarities to rhodopsin-like GPCRs. Together, these findings identify a host-dependent peptide–GPCR signaling system that links plant protease activity to fungal developmental reprogramming during infection.

## Results

### The GPCR Gpe1 is activated by a Pit2-derived peptide

To systematically explore GPCRs in *U. maydis*, we performed a genome-wide search for proteins containing the canonical seven-transmembrane (7TM) architecture using a structure-based approach utilizing Alphafold2^18^ (see Materials and “Methods” section for details). This analysis identified 34 putative GPCRs (Supplementary Data 1), of which only nine had been suggested by previous studies^2,19,20^. Among the newly identified, one candidate (UMAG_01374) was reported as a crucial virulence factor^11^, previously designated as protein important for tumors 1 (Pit1)^11^, which we renamed to G-protein-coupled receptor promoting fungal *in planta* expansion 1 (Gpe1).

**Fig. 1:**
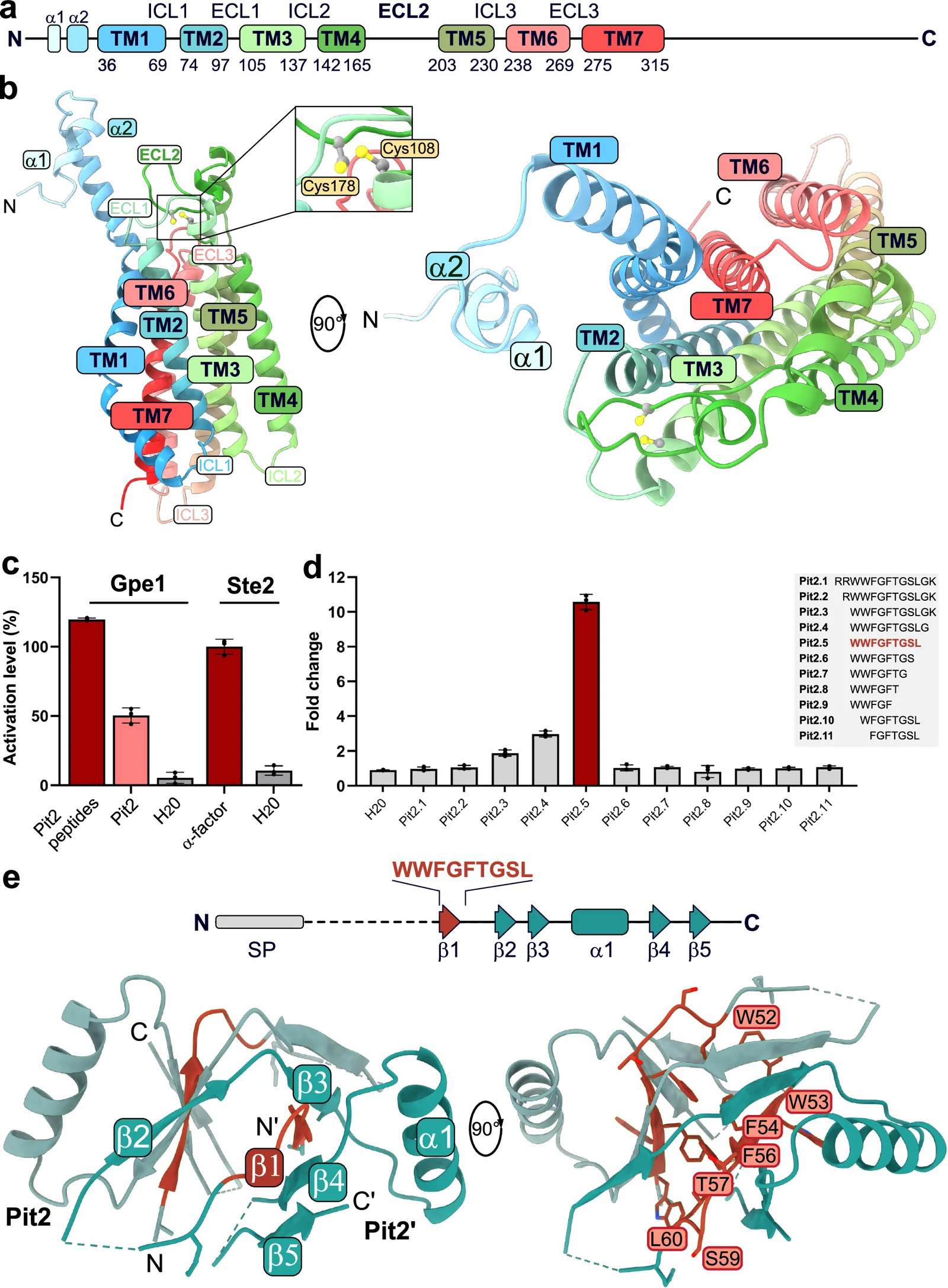
Structural and functional analysis of the GPCR Gpe1 and its Pit2 peptide ligand. **a** Schematic representation of the domain architecture of Gpe1, indicating predicted transmembrane (TM) helices, intracellular (ICL) and extracellular loops (ECL), and N-/C-terminal regions. Colored in rainbow from N- (blue) to C-terminus (red). **b** Side and top views of the predicted Gpe1 monomer structure with TM helices and loops labeled. A potential disulfide bond between Cys108 and Cys178 stabilizing the extracellular region is highlighted. Cartoon is colored in rainbow from N- (blue) to C-terminus (red). **c** Activation of Gpe1 by 100 µM full-length Pit2, 10 µM Pit2 peptides and *S. cerevisiae* Ste2 by 10 µM α-factor through fluorescent readout. Fluorescence levels are normalized to the Ste2 activation being 100 %. **d** Fold-change activation of Gpe1 by a panel of self-secreted peptide variants (Pit2.1 to Pit2.11), with Pit2.5 (WWFGFTGSL) showing the strongest activation. Peptide sequences are listed on the right. **e** Schematic of the Pit2 precursor protein, including its signal peptide (SP) and the position of the activating peptide (WWFGFTGSL) within the structural elements (β-strands and α-helices) (top). Crystal structure of the Pit2 homodimer (bottom) with protomers colored in cyan and light cyan. Secondary structure elements are labeled, and the residues of the activating peptide buried in the dimer core highlighted in red. Activation level and fold change values are presented as the mean of three independent biological experiments with standard deviation (SD). Source data from the fluorescence-based yeast reporter assays are provided as a Source Data file.

Gpe1 is a 435-amino acid protein, featuring a short (35 aa) extracellular N-terminus and a longer (120 aa) unstructured intracellular C-terminus (Fig. 1a and Supplementary Fig. 1a). Three extra- and intracellular loops connect the TMs, with the N-terminal 35 residues and the 38 residues of the second extracellular loop (ECL2) forming an extracellular extension (Fig. 1b). ECL2 is also predicted to be connected to TM3 via a disulfide bond formed by Cys178 and Cys108 (Fig. 1b), a hallmark of many rhodopsin-class GPCRs^21^. To enable ligand identification, Gpe1 was then expressed in an engineered *Saccharomyces cerevisiae* strain to test receptor activation through production of a fluorescent reporter^22^. This strain is devoid of both endogenous mating GPCR genes (STE2 and STE3), all pheromone genes (MFA1/2 and MFALPHA1/MFALPHA2), BAR1, SST2 and FAR1^22^. And carries an unmodified version of ScGPA1, which is the homolog of *U. maydis* Gpa1^23^. The coupling of ScGPA1 with Gpe1, and later MAPK pathway activation, lead to the induction of a red fluorescent protein (yEmRFP) under control of the FUS1 promoter serves as readout. Western blot analysis confirmed expression of Gpe1 in *S. cerevisiae* (Supplementary Fig.1b).

The neighboring gene of *gpe1* encodes the secreted effector Pit2, which is cleaved by maize papain-like cysteine proteases (PLCPs) to release a protease-inhibitory peptide^12,13^. Deletion of *gpe1* or *pit2* causes a phenotypically identical reduction in virulence, although no functional link between both proteins has been established^11^. We hypothesized that Pit2 might act as or contain the activating ligand of Gpe1, and we therefore purified recombinant Pit2 lacking its signal peptide (Supplementary Fig. 2a). Purified Pit2 was applied to yeast expressing Gpe1, inducing a reporter response reaching ~ 25%. ScSte2 with α-pheromone as a full agonist serves as a positive control (Fig. 1c). Because Pit2 is cleaved by maize PLCPs, we next tested whether cleavage enhances activity. Remarkably, tryptic digestion of Pit2 peptides - resulting in a similar cleavage pattern compared to PLCP treatment^12,13^ - increased the reporter signal to ~100% at tenfold lower peptide concentration (Fig. 1c), demonstrating robust Gpe1 activation by processed Pit2 fragments in *S. cerevisiae*. Reporter response, therefore, demonstrates a functional interaction of Gpe1 with ScGpa1. Comparative structure predictions with the *U. maydis* Gα subunits suggest UmGpa1 as a candidate interaction partner for Gpe1; however, this hypothesis and the predicted interaction geometry require experimental validation (Supplementary Fig. 3).

Peptide fractionation and mass spectrometry identified an 11–amino acid Pit2 fragment (WWFGFTGSLGK) as a Gpe1 agonist (Supplementary Fig. 2b, c). To delineate the core activation motif, we employed a yeast biosensor system featuring peptide secretion and monitoring of GPCR activation^22^. This analysis mapped the core sequence to WWFGFTGSL (Pit2.5), triggering a nearly tenfold increase in fluorescence compared to the 11-amino acid sequence motif (Fig. 1d). Intriguingly, the presence of an N-terminal RR-motif suppressed Gpe1 activation. Previous work had implicated a 14-residue, RR-containing Pit2 peptide (PID14) as PLCP-derived protease inhibitor^12,13^. Consistently, the shorter WWFGFTGSL peptide also arises from PLCP cleavage but lacks protease-inhibitory activity^12^. Together, these findings establish Gpe1 as a dedicated receptor for a Pit2-derived peptide, with activation strictly depending on host PLCP processing.

### Pit2 serves as scaffold for the Gpe1-activating peptide

Both, Gpe1 and Pit2 are crucial for plant infection and subjected to tight regulation on the transcriptional and post-transcriptional level^24,25^. Another layer of regulation seems to be contained in the Pit2 protein, which protects and hides the activating peptide prior to host-dependent activation. Consistent with that, Pit2 is secreted in full-length by *U. maydis* without evidence of endogenous cleavage^24^. Size-exclusion chromatography of Pit2 further supported a stable protein scaffold, revealing a single monodisperse fraction (Supplementary Fig. 2a). To understand the precise nature of this protein chassis, we crystallized Pit2 and solved its crystal structure at 2.3 Å resolution (Supplementary Data 2). Residues 51 to 120 could be unambiguously placed in the electron density except for the N-terminus and flexible regions decorating the protein core. Pit2 is composed of five β-strands and one α-helix, forming a tightly intertwined homodimer. The dimer interface buries the conserved hydrophobic WWFGFTGSL motif forming β1 with W52, W53, F54, and F56 contributing to the hydrophobic packing (Fig. 1g). Strands β3-5 of one protomer and β2 from the neighboring Pit2 molecule complete the dimer interface (Fig. 1e and Supplementary Fig. 4). Our results indicate that Pit2 acts as a structural chassis containing the activating peptide in the homodimer interface, thereby preventing premature activation of Gpe1 prior to host-mediated proteolytic release of Pit2-derived peptides.

### Conserved residues at Gpe1 are required for Pit2 peptide binding

**Fig. 2:**
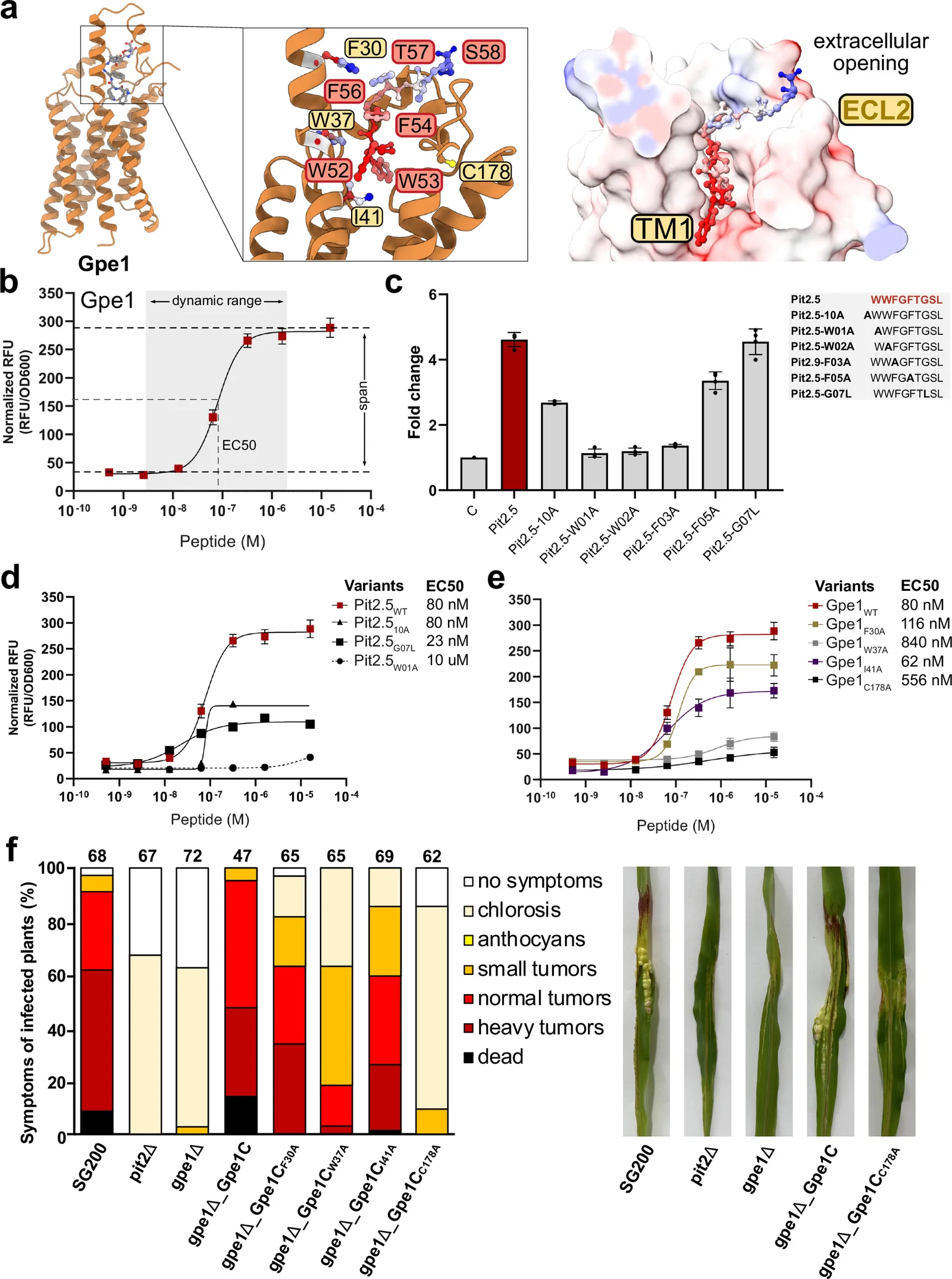
Structural determinants of Pit2 peptide binding and functional relevance of Gpe1. **a** Predicted binding mode of the Pit2-derived peptide to Gpe1, highlighting residues at the interface (left). Close-up view shows aromatic and hydrophobic residues in TM1 and ECL2 (middle), with surface representation illustrating the extracellular opening of the binding pocket (right). **b** Dose–response curve of wild-type Gpe1 activation by the Pit2 core peptide (WWFGFTGSL), showing normalized fluorescence reporter units (RFU) as a function of peptide concentration. The shaded area indicates dynamic range, with EC_50_ marked. **c** Fold change activation of Gpe1 using different Pit2.5 peptide versions. **d** Dose-response curves of selected Pit2.5 peptide versions with EC_50_ values given on the right. **e** Dose–response curves of Gpe1 variants mutated at predicted binding site residues, compared to wild-type Gpe1. Right: table summarizing EC_50_ values for the indicated variants. **f** Maize seedling infection assays using *U. maydis* SG200 strains and derivatives thereof. Disease symptoms were rated 12 days after inoculation (dpi) and grouped into categories as shown in the legend. Numbers of infected plants are given above each column, representing three biological replicates. Images show representative infection symptoms. Fold change and normalized RFU values are presented as the mean of three independent biological experiments with SD as error bars. Source data from the fluorescence-based yeast reporter assays are provided as a Source Data file.

To investigate the structural basis of Gpe1 activation, we modeled Gpe1-Pit2 complexes using Alphafold3. As AlphaFold-based predictions, particularly for peptide-bound GPCR complexes, may not accurately reflect the true binding mode, we considered these predictions only to guide mutagenesis. In the predicted structure, the N-terminal tryptophan residues of the Pit2 peptide are buried top-down in a cavity formed by TM1 and ECL2 of Gpe1, with the C-terminus oriented extracellularly (Fig. 2a). This binding mode is also supported by our peptide truncation experiments, where C-terminal extensions of the Pit2 peptide still showed activation of Gpe1 (Fig. 1d, Pit2.3, Pit2.4), while N-terminal extensions or deletions did not. To further substantiate this potential Pit2 peptide binding mode to Gpe1, we generated different peptide versions based on the Pit2.5 agonist. Dose-response analysis revealed an EC_50_ of 80 nM for wildtype Gpe1 (Fig. 2b), consistent with values reported for fungal Ste2 pheromone peptide receptors^22,26^. Substitution of the first three N-terminal hydrophobic residues strongly reduced activation, indicating that this region is critical for the peptide engagement with the Gpe1 binding pocket (Fig. 2c). Even the addition of a single alanine residue in the N-terminal site reduced Gpe1 activation significantly (Fig. 2c). In contrast, residue substitutions closer to the C-terminus had only moderate effects on Gpe1 activation (Fig. 2c). Dose response analysis on selected peptide variants corroborate these observations (Fig. 2d). The peptide mutant Pit2.5-G07L showed a higher apparent potency, with an EC_50_ of 23 nM, but reduced maximum fluorescence. On the other hand, the Pit2.5-W01A substitution abolished receptor activation (Fig. 2d). Notably, while addition of an N-terminal alanine residue did not strongly affect EC_50_, maximum fluorescence was strongly decreased. Notably, higher concentrations of this peptide impaired the growth of *S. cerevisiae*. Together, these data highlight the strong sequence sensitivity of the Gpe1/Pit2 interaction and suggest that minor modifications, especially in the N-terminus of the agonist peptide, strongly affect Gpe1 activation.

Advised by the structure predictions, we further investigated how the variation of residues at Gpe1 in vicinity of the predicted peptide binding pocket would influence GPCR activation (Fig. 2e). While some mutants (F30A and I41A) only moderately influenced EC_50_, dynamic range and maximum activation levels, variation of C178A and W37A strongly decreased EC_50_ and rendered Gpe1 almost non-responsive (Fig. 2e and Supplementary Fig. 5).

Having established the in vitro importance of these residues, we next tested their functional relevance *in planta*. Deletion strains of *gpe1* were complemented with the different *gpe1* mutant alleles, and maize infection assays were performed to assess virulence (Fig. 2f and Supplementary Data 3). As reported earlier, deletion of *gpe1* or *pit2* almost completely abolishes virulence, preventing tumor formation (Fig. 2f)^11^. Complementation with the native *gpe1* and *gpe1*_*F30A*_ restored virulence to wildtype levels, while strains producing the Gpe1_I41A_ variant show a slight decrease and strains containing C178A and W37A a strong attenuation in virulence, following the results obtained from in vitro experiments (Fig. 2e, f). Collectively, our structure-guided mutational analysis confirmed the functional relevance of the Pit2 coordination by Gpe1 and revealed that disrupting this interaction strongly influences the virulence of *U. maydis*.

### Pit2-mediated activation of Gpe1 induces receptor endocytosis and promotes proliferation

Gpe1 and Pit2 are important regulators of fungal fitness during plant infection, but the precise physiological impact is still unclear. To assess whether and how Gpe1 activation modulates fungal physiology, we constitutively expressed a green fluorescently tagged version of Gpe1 (Gpe1-G) in the haploid pathogenic *U. maydis* strain SG200.

**Fig. 3:**
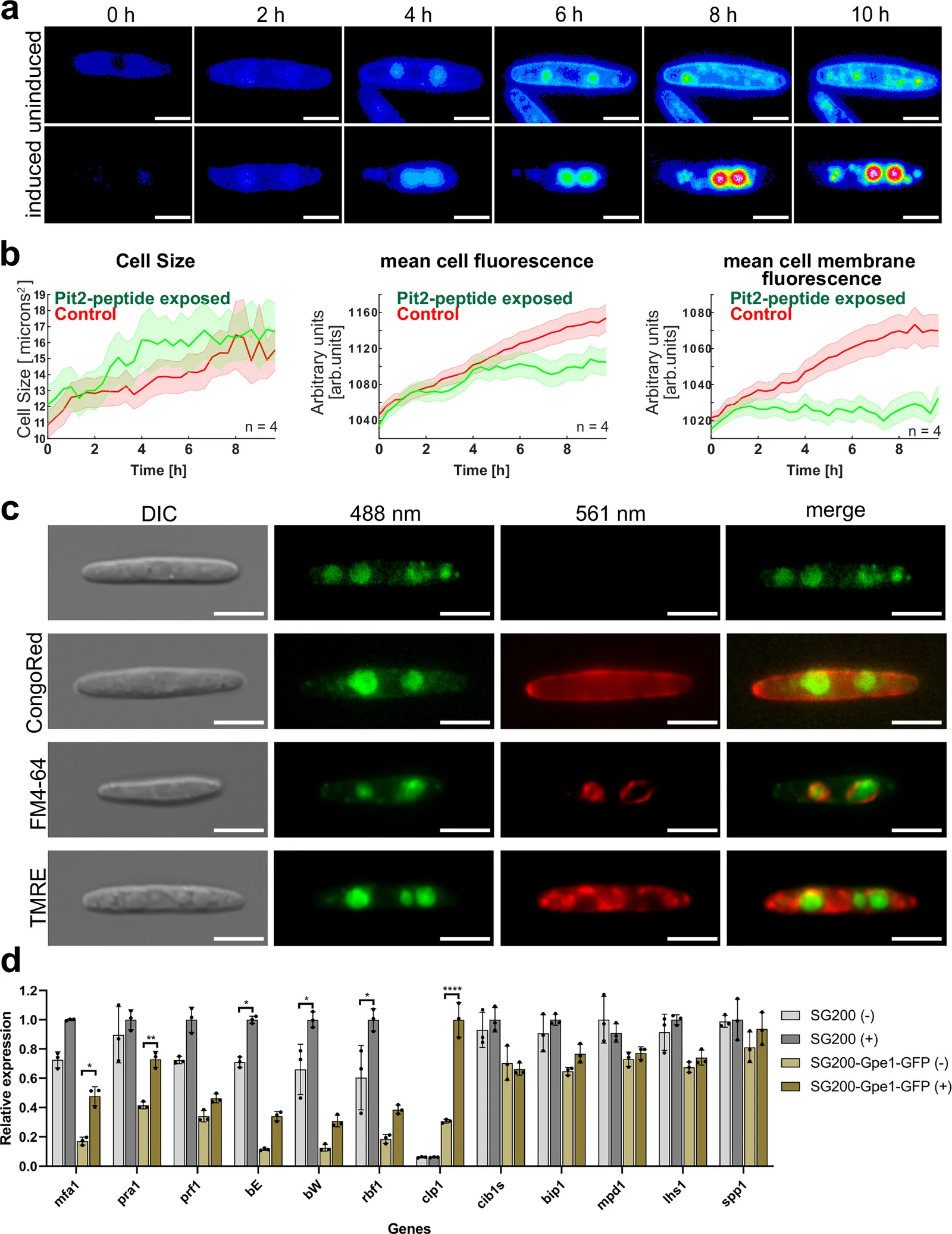
Gpe1 activation by Pit2 results in receptor internalization and promotes proliferation of *U. maydis*. **a** Time-resolved imaging of Gpe1-G cells under uninduced or Pit2 peptide induced conditions using a microfluidic microscopy setup. Heat maps show fluorescence intensities at the indicated time points. The experiments were repeated independently three times with similar results. **b** Quantitative analysis of cell size and mean cellular or membrane-associated fluorescence over time. Data is represented as mean with standard error of the mean (SEM) from three independent biological experiments (green, induced; red, uninduced). **c** Subcellular localization of Gpe1-G in induced cells stained with Congo Red (fungal cell wall), FM4-64 (vacuolar membrane) and TMRE (mitochondrial membrane). Differential interference contrast (DIC), single-channel images with 488 nm or 561 nm excitation, and merged images are shown. Scale bars correspond to 10 μm. The experiments were repeated independently three times with similar results. **d** qRT-PCR of mating type and UPR marker genes in SG200 WT and Gpe1-G expressing cells with (+) and without (-) Pit2 peptide stimulation. Error bars are represented with the SD of three biological replicates. eIF2b was used for normalization of gene expression. Two-way ANOVA followed by Tukey was used to compare SG200 WT and Gpe1-G expressing cells with (+) and without (-) Pit2 peptide stimulation with significance at * (*p* < 0.05), ** (*p* < 0.01) and **** (*p* < 0.0001). Comparisons not marked with an asterisk were considered not statistically significant (ns; *p* > 0.05). Source data from the fluorescence-based yeast reporter assays are provided as a Source Data file.

Sporidial cells of the strain were subjected to a microfluidic microscopy setup that enables continuous incubation, live-cell imaging and controlled perfusion of the Pit2 peptide. In the absence of exogenous peptide, Gpe1-G displayed a plasma membrane-associated localization but also localized to vesicular structures (Fig. 3a, b), supporting earlier observations^11^. Upon addition of activating Pit2 peptide, rapid internalization of Gpe1-G into the endomembrane system was observed, suggesting ligand-induced endocytosis and receptor recycling (Fig. 3a, b).

To further characterize the subcellular compartmentalization of internalized Gpe1, we performed co-localization experiments using organelle-specific dyes (Fig. 3c). Vacuolar staining with FM4-64 showed that Gpe1-G mostly localizes to vacuoles, suggesting that the majority of internalized Gpe1 is trafficked to the vacuole for degradation (Fig. 3c). These findings indicate that Pit2 stimulation triggers ligand-dependent endocytic trafficking of Gpe1, followed by receptor turnover via the vacuolar degradation pathway.

Notably, we also observed that the Gpe1-G strain reacted with elevated growth rates (Supplementary Movies 1 and 2). Upon Pit2 peptide exposure, individual fungal cells divided more rapidly, suggesting an accelerated cell cycle progression (Supplementary Fig. 6a, b). More precisely, the time required to complete a cell division cycle (from one budding to the next budding event) decreased significantly from 226±40 min to 167 ± 10 min upon treatment with Pit2 peptide (Supplementary Data 4). In *U. maydis*, the infection process requires a complex transcriptional program inducing a G2 cell cycle arrest during mating, prioritizing polar growth during the early stage of plant colonization^27^ (Supplementary Fig. 7). The transcription factors bE/bW and Rbf1 control polar growth and cell cycle arrest^27,28^ and the regulatory protein Clp1 has been implicated to play a decisive role in the proliferation vs. cell cycle arrest process^29^. *In planta*, the cell cycle arrest is released, and proliferation is prioritized again to enable massive fungal growth followed by gall formation. Clp1 exhibits extensive crosstalk with the central Hac1-like UPR regulator Cib1 at the onset of biotrophic growth *in planta*^30^. Cib1 directly regulates the expression of pathogenicity genes, including *gpe1* (*pit1*) and *pit2*^24^. Our observation that activation of Gpe1 strongly promotes proliferation now suggests that activated Gpe1 potentially acts via Clp1 and/or Cib1 (Supplementary Fig. 7). To test this hypothesis, we treated SG200 WT and Gpe1-G expressing cells with the Pit2 peptide and determined gene expression changes of mating type and UPR marker genes by qRT-PCR. In comparison to WT cells, constitutive expression of Gpe1 decreased transcript levels of all tested mating type pathway associated genes with the notable exception of *clp1*. Transcript levels were approximately 5-fold higher in the Gpe1 expressing strain (Fig. 3d and Supplementary Data 5). Strikingly, Pit2 peptide further increased expression of *clp1* in the Gpe1 expressing background, but not in the SG200 control (Fig. 3d). All other mating type-associated genes showed slightly increased transcript levels after Pit2 peptide treatment and UPR marker genes were not affected by Gpe1 expression or Pit2 peptide. These data suggest that Pit2 peptide-dependent activation of Gpe1 preferentially promotes *clp1* expression rather than inducing a broad mating type or UPR transcriptional response under these conditions. Re-initiation of the fungal cell cycle after successful plant penetration is mediated by the physical interaction between Clp1 and Cib1, which promotes mutual stabilization, Clp1 accumulation and modified UPR signaling^30,31^. Increased Clp1 protein levels establish a negative feedback loop on the mating type pathway, which contributes to cell cycle re-initiation^27,29^. Hence, although we cannot exclude that posttranscriptional regulation of Clp1 is involved in Pit2-dependent re-initiation of growth, increased *clp1* transcript levels after Pit2 peptide-mediated Gpe1 activation provide a plausible mechanistic link between host-dependent peptide perception and re-initiation of fungal proliferation.

### The Gpe1/Pit2 shows co-evolutionary signatures in smut fungi and similarities to mammalian rhodopsin receptors

**Fig. 4:**
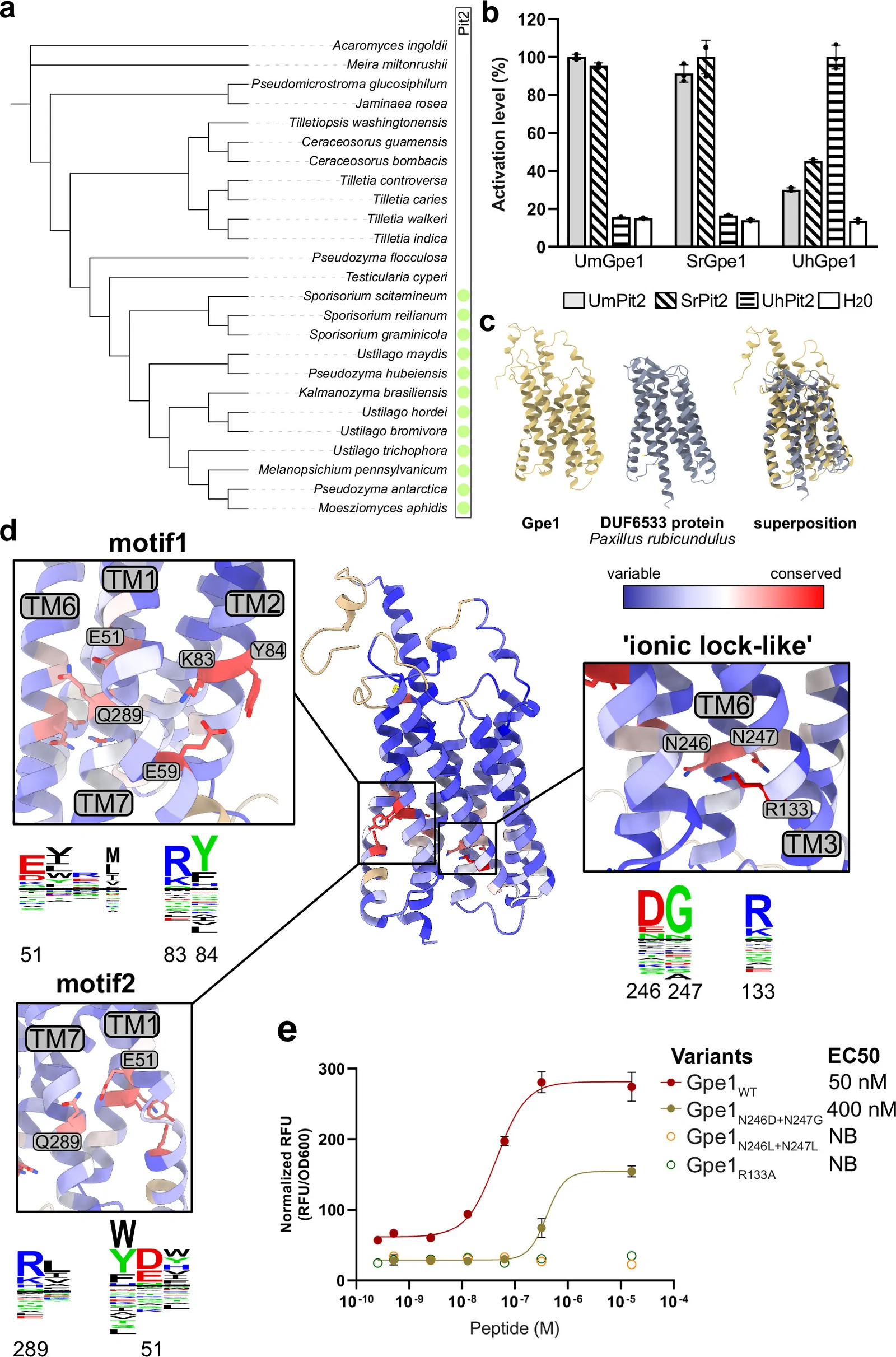
Role and co-evolution of the Pit2–Gpe1 system A. **a** Phylogenetic distribution of Gpe1 and Pit2 across smut fungi. Presence of Pit2 is indicated by a green colored circle. **b** Activation of Gpe1 homologs from *Ustilago maydis* (UmGpe1), *Sporisorium reilianum* (SrGpe1), and *Ustilago hordei* (UhGpe1) by the corresponding Pit2 peptides measured in *S. cerevisiae* biosensor assays. Activation level values are presented as the mean of three independent biological experiments with SD. **c** Structural models of Gpe1, a DUF6533 protein from *Paxillus rubicundulus* (A0A0D0C6P1) and superposition of the two. **d** Predicted structural model of Gpe1 colored according to sequence conservation, with blue showing variable and red showing conserved residues. Three motifs are highlighted in the respective boxes. **e** Dose-response curves of Gpe1 with variations in the ionic-lock like motif, compared to wild-type Gpe1. Right: table summarizing EC_50_ values for the indicated variants. NB, No binding. Source data from the fluorescence-based yeast reporter assays are provided as a Source Data file.

Gpe1 homologs are present in many smut fungi, whereas Pit2 is restricted to species closely related to *U. maydis* (Fig. 4a and Supplementary Data 6). Although Pit2 sequences display little overall conservation, the activating peptide residues are highly conserved (Supplementary Fig. 8). To test cross-compatibility, we analyzed Pit2 homologs from *S. reilianum* (SrPit2) and *U. hordei* (UhPit2). Peptides corresponding to the *U. maydis* activating motif were assayed with the respective Gpe1 homologs in *S. cerevisiae*. SrPit2 activated UmGpe1 to levels comparable with UmPit2, whereas UhPit2 failed to do so (Fig. 4b). Conversely, UmPit2 and SrPit2 only weakly stimulated UhGpe1 (30% and 45% activity, respectively; Fig. 4b). These reciprocal activities suggest co-evolution of Gpe1 and Pit2, where minor sequence differences reduce cross-activation (Fig. 4b).

To investigate a potential conservation of the Gpe1 system beyond smut fungi, we performed a structure based homology search based on the Alphafold model of Gpe1 using Foldseek^32^ and restricted to fungi. This analysis identified a large number of fungal GPCRs from diverse species all belonging to basidiomycetes (Supplementary Data 7). Many of these proteins are characterized by a domain of unknown function (DUF6533) and the closest structural homolog from *Paxillus rubicundulus* had a TM-score of 0.8 and an r.m.s.d. of 4 Å, indicating some structural similarity and a shared global fold (Fig. 4c). This suggests that Gpe1 is part of a broader, evolutionary conserved receptor family. To further investigate the similarities among the identified GPCRs and Gpe1, we used FoldMason^33^ to perform a multiple structural alignment and systematically map structurally conserved residues across the receptor family. Strikingly, conservation is not uniformly distributed across the 7TM bundle but instead clusters into three discrete intramolecular patches, each positioned at potentially functionally strategic interfaces within the helical core (Fig. 4d).

The first conserved patch is located at the TM1–TM2–TM7 interface (Fig. 4d, motif 1). Here, residues corresponding to E59 (TM1), K83 and Y84 (TM2) form a compact interaction network. These residues are highly conserved at the sequence level and positioned in a way that might stabilize helix packing between the extracellular halves of TM1 and TM2. The second conserved patch reinforces contacts between TM1 and TM7 (motif 2), involving E51 and Q289 (Fig. 4d, motif 2). The third conserved patch is centered on TM3 and TM6. Specifically, a conserved basic residue at position 3.50 (R133) is positioned to interact with conserved polar residues in TM6 (N246 and N247). Notably, the NN sequence seems to be unique to Gpe1, while all other identified GPCRs have a DG within TM6 (Fig. 4d). Overall, this residue network is reminiscent of the ionic lock described in rhodopsin/class A GPCRs, where the R3.50-containing motif stabilizes the inactive state and undergoes rearrangement during activation^34,35^. However, dissimilar to the ionic lock, no intrahelical contact seems to stabilize R133 in Gpe1.

To test the functional relevance of this putative TM3–TM6 activation module, we generated Gpe1 variants targeting R133 and the adjacent TM6 residues N246 and N247 (Supplementary Fig. 5). Gpe1 variant activation was analyzed by the yeast reporter system. Wild-type Gpe1 responded robustly to the peptide, while substitution of N246D + N247G, strongly reduced receptor activation and shifted the apparent EC_50_ to approximately 400 nM, while also decreasing the maximum reporter response. More disruptive substitutions, N246L + N247L and R133A, abolished detectable receptor activation. These results suggest that both the conserved R3.50 residue and the TM6 NN motif are required for efficient Gpe1 activation. However, reduced activity of individual variants may also reflect effects on receptor stability or intracellular trafficking competence.

Collectively, the structural conservation of this region suggests a potential evolutionary connection to rhodopsin/class A-like GPCR activation principles, despite clear differences.

**Fig. 5:**
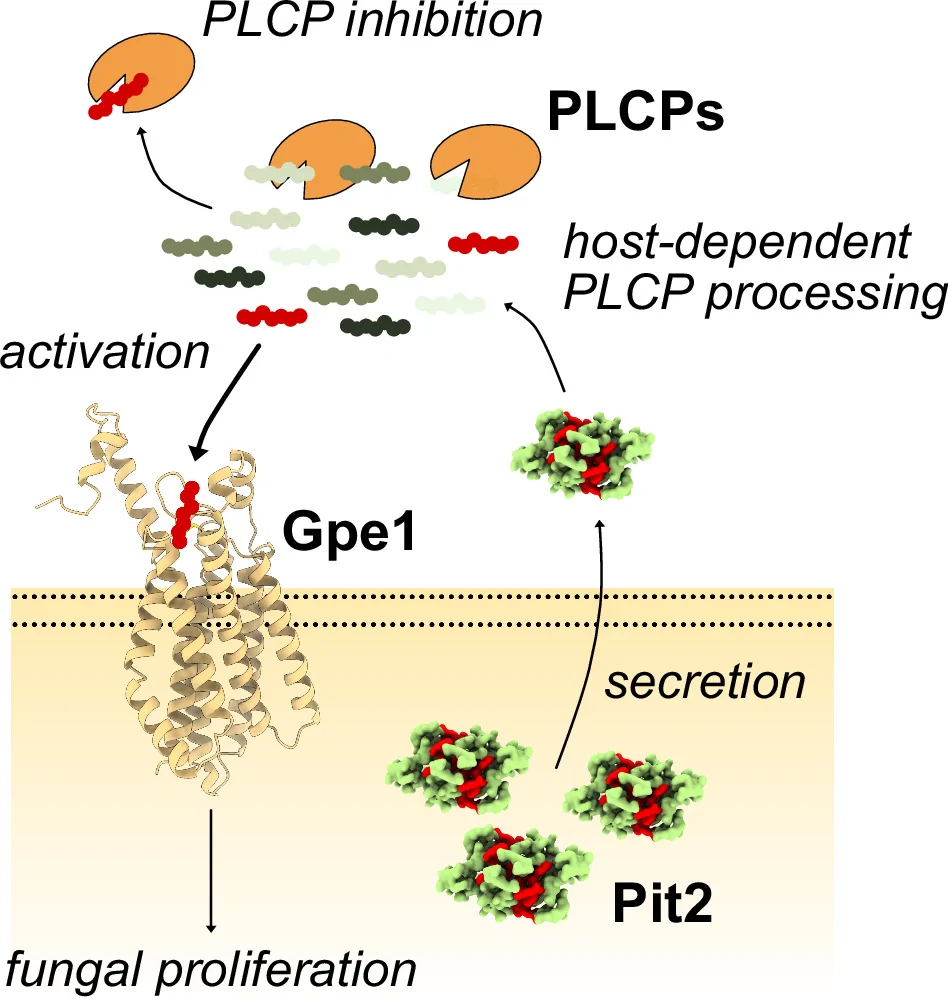
Schematic model of Gpe1-Pit2 function. Secreted Pit2 is processed by host papain-like cysteine proteases (PLCPs), generating peptides that activate Gpe1 and inhibit the PLCPs. Gpe1 activation triggers fungal proliferation.

In conclusion, our work identified a host-dependent peptide–GPCR signaling system in smut fungi, mediating fungal recognition of the host-specific plant environment through proteolytic activity of host proteases. Several hundred proteases regulate central developmental programs in plants^36^, and more recent studies highlight their central role in immunity^37,38^. Especially, PLCPs are broadly conserved and often strongly induced in different plant tissues upon pathogen infections and stresses^38^. The Gpe1/Pit2 system, therefore, hijacks a central and indispensable aspect of plant immunity, potentially balancing immune suppression with fungal proliferation *in planta*^39^ (Fig. 5). This is supported by the fact that PLCP cleavage generates different peptides species^12^ that either inhibit PLCP activity or activate Gpe1 to promote fungal proliferation. Future work has to investigate the individual contribution of these two peptide versions during *U. maydis* infection.

Our study furthermore expands the repertoire of peptide-based communication systems in fungi. In addition to the pheromone mating system, where endogenous peptides are secreted and processed by fungal enzymes^40^, Pit2 cleavage to release the ‘hidden’ activating peptide is host dependent^12,13^. Hence, similar host-gated signaling modules may be more widespread in pathogenic and symbiotic fungi than previously recognized. From an evolutionary perspective, the parallels between Pit2-derived signaling and fungal pheromone systems raise the possibility that fungi have repeatedly co-opted short, protease-processed peptides as ligands for GPCRs, tailoring these systems to different ecological contexts. Exploring peptide-GPCR pairs across fungal lineages may therefore uncover a hidden communication network that integrates fungal life stages within microbial communities and host environments.

## Methods

### Accession numbers

*U. maydis, S. reilianum* and *U. hordei* Gpe1-Pit2 sequences were obtained from FungiDB (https://fungidb.org/fungidb/app/): UmGpe1 (UMAG_01374), UmPit2 (UMAG_01375), SrGpe1 (sr10528), SrPit2 (sr10529), UhGpe1 (UHOR_02063) and UhPit2 (UHOR_02064).

### Strains and growth conditions

All strains used and generated in this study are listed in Supplementary Data 8. The *Escherichia coli* strain TOP10 (Thermo Fisher Scientific) was used for cloning purposes. The *E. coli* strain SHuffle® (DE3) (Novagen) was used to express all produced proteins in this study. *E. coli* strains were grown in dYT medium (1.6% (w/v) Bacto-Tryptone, 1 % (w/v) yeast extract and 0.5% (w/v) sodium chloride) or Luria Broth (LB) media, supplemented with chloramphenicol (50 µg/mL) or ampicillin (100 µg/mL) under constant shaking (200 RPM) in a temperature-controlled incubator at 37 °C or on dYT-agar at 37 °C. *Zea mays* cv. Early Golden Bantam (EGB, Urban Farmer, Westfield, IN, USA) was used for infection assays with *Ustilago maydis* and grown in a temperature-controlled greenhouse (light and dark cycles of 14 hours at 28 °C and 10 h at 20 °C, respectively). *U. maydis* strains were grown in YEPSlight medium (1 % (w/v) yeast extract, 0.4% (w/v) peptone and 0.4% (w/v) sucrose) at 28 °C with baffled flasks under constant shaking at 250 RPM or on potato dextrose-agar at 28 °C. *Saccharomyces cerevisiae* strain *JTy014* was grown in Synthetic Complete (SC) media (all amino acids and 2% (w/v) glucose, pH 6.5) or on potato dextrose-agar at 28 °C. *S. cerevisiae* transformants, which express GPCRs were grown in Synthetic dropout media (SD) media (SC media lacking uracil), Yeast Nitrogen Base (YNB) media (plus 0.01 % (w/v) methionine, 0.008 % (w/v) histidine, 0.04 % (w/v) leucine and 2% (w/v) glucose, pH 6.5) for fluorescence measurements or SD-agar at 28 °C. *S. cerevisiae* transformants with peptide secretion vectors were grown in synthetic dropout media (SC media lacking uracil and histidine) media with 1% sucrose instead of glucose (1% galactose was added to induce peptide expression).

### DNA amplification and molecular cloning

All plasmids and oligonucleotides used in this study are listed Supplementary Data 8. Primers were synthetized by Merck KGaA. The open reading frames of all effectors cloned in this study were amplified from the genomic DNA of *U. maydis*, *S. reilianum* and *U. hordei*. All genes were amplified without their predicted signal peptide (SignalP 5.0) (Almagro Armenteros et al., 2019) employing specific oligonucleotides. A standard PCR protocol with Q5® High Fidelity DNA polymerase (New England Biolabs) and primer-specific annealing temperatures was used for the DNA amplification.

For the plasmid constructions, standard molecular cloning strategies and techniques were applied. For GPCR expression in *S. cerevisiae*, the ScSte2 expression vector pRS416^22^ was digested with *SpeI* and *XhoI* to remove the ScSte2 gene. The pure backbone vector was used for classical restriction-ligation (T4 ligase) to clone the GPCR genes inside using unique restriction sites (*SpeI* and *XhoI*) added in the PCR gene amplification. For plasmid amplification, recombinant plasmids were transformed in chemically competent *E. coli* Top10 cells. For site directed mutagenesis in the *gpe1* wild-type gene, whole-plasmid amplifications were performed using a standard PCR protocol with Q5® High Fidelity DNA polymerase (New England Biolabs), primers carrying a different codon, for example alanine, instead the targeted amino acid, and as a DNA template the UmGpe1 expression vector (pIL0186).

Multiple plasmids from the MoClo yeast toolkit^41^ were used to construct peptide secretion vectors. The GFP-dropout fragment was PCR-amplified to add *BsmBI* sites at both ends, followed by digestion with *BsmBI* (NEB). The secretion cassette of α-factor was obtained by amplifying pSB21 using primer ML215 and ML216. The YTK plasmids and pre-pro region PCR products were first assembled using the Golden Gate system, then ligated with BsmBI-digested GFP-dropout product to form the final plasmid. All Pit2 variants were synthesized as single-stranded oligonucleotides, and double-stranded DNA was generated either by PCR using a reverse primer SB320 or by annealing complementary primers. The resulting DNA fragments were inserted into the secretion vector using Golden Gate assembly. A GAL1 promoter was used to drive peptide expression.

For cloning of protein expression constructs, the pEM-GB1 vector was used for, encoding the solubility tag GB1, a hexahistidine (6His) tag and a *Tobacco etch virus* protease (TEV) recognition site. Plasmid assembly was performed by Golden Gate assembly, the backbone vector and gene amplicon were used for classical one-pot BsaI restriction-ligation (T4 ligase) to form the final plasmid^42^. All plasmids were verified by Sanger sequencing.

### Transformation and generation of *S. cerevisiae* strains

A main culture of the respective *S. cerevisiae* ancestor strain was inoculated with an overnight preculture at a starting OD_600_ of 0.15 and incubated at 28 °C for 3-5 h. Subsequently, the main culture was harvested and washed with sterile water. Washed cells were resuspended in 1 mL of 100 mM of lithium acetate. Cells were transferred to 1.5 mL centrifuge tubes, harvested again and resuspended now in 0.1 mL of 100 mM of lithium acetate per transformant (0.5 mL for 5 transformants). For each transformant, 0.1 mL of resuspended cells were transfer in a new 1.5 mL centrifuge tubes, centrifuged and the following solutions were added in this order: 240 μL of 50 % (w/v) PEG 3350, 36 μL of 1 M lithium acetate, 20 μL of boiled carrier DNA (salmon testes DNA, 5 mg/mL), 2 μL ( ~ 2 μg) of plasmid (except for the negative control which sterile water was used instead) and necessary sterile water until a final volume of 360 μL. The transformation reaction was vortexed vigorously until the cell pellet was completely resuspended. Then, two incubation steps were performed without shaking: 30 °C for 30 min and 42 °C for 35 min. After a quick centrifugation, the supernatant was discarded, and the cells were resuspended in 400 μL of sterile water smoothly using the micropipette. Onto two selection plates (SD-agar), the cells were streaked, one plate with 100 μL and the second one with 300 μL. The plates were incubated at 28 °C for 2-3 days.

### Western blotting

*S. cerevisiae* strains were grown in 5 ml SC-Ura medium overnight. Cells were harvested at 500 x *g* for 2 min and resuspended in 250 µl cold 1 x PBS + cOmplete™ Protease Inhibitor Cocktail (Roche, CO-RO). Glass beads were added before processing in a Mixer Mill MM 400 (Retsch GmbH, Haan, Germany) at 30 Hz for 2 rounds of 5 minutes each. Cell debris was harvested at 500 x *g* for 5 min at 4 °C. The supernatant was then centrifuged at 16000 x *g* for 15 min at 4 °C, and the membrane fraction containing pellets were resuspended in 50 µl cold 1x PBS + cOmplete™ Protease Inhibitor Cocktail. 50 µl of the sample was mixed with 50 µl 5*x* Laemmli buffer (62.5 mM Tris-HCl, 2% SDS, 10% glycerol, 0.01% bromophenol blue, 5% β-mercaptoethanol, pH 6.8) and incubated at 50 °C for 10 min.

10 µl of the sample was loaded onto a 12% SDS-PAGE gel and run at 100 V for 10 min followed by 180 *V* for 40 min. Blotting is performed using PVDF membrane at 45 mA for 1 h, with subsequent blocking in TBST (20 mM Tris-HCl, 150 mM NaCl, 0.1% Tween-20, pH 7.4–7.6) + 3% milk powder overnight. The membrane was then incubated with 1:6000 Mouse Anti-HA antibody (Roche Diagnostics GmbH, Mannheim, Germany) for 2.5 h, washed three times with TBST and then incubated with Anti-Mouse IgG-HRP antibody (Promega Corporation, Madison, WI, USA) for 1.5 h. Following three washes with TBS (20 mM Tris-HCl, 150 mM NaCl, pH 7.4–7.6), the membrane was incubated with ECL Prime (Cytiva, Marlborough, MA, USA) for 1 minute. Detection was performed using an ImageQuant LAS4000 (Cytiva, Marlborough, MA, USA).

### Protein production and purification

Plasmids were transformed into *E. coli* strain BL21 (DE3) (Novagen). Expression test conditions were induced in dYT median supplemented with the required antibiotic and lactose 1% for 20 h at 28 °C, IPTG 0.5 mM for 3 h at 37 °C or IPTG 0.5 mM for 20 h at 20 °C, respectively at 200 RPM. After protein expression at the best condition, the cells were harvest (3993 x *g*, 15 min, 4 °C), washed, resuspended in lysis buffer (20 mM HEPES pH 8, 20 mM KCl, 250 mM NaCl and 40 mM imidazole) and lysate using a microfluidizer (M110-L, Microfluidics). All *E. coli* debris were collected by centrifugation (48 384 x *g*, 15 min, 4 °C) and membrane free supernatant was loaded onto Ni-NTA FF-His Trap columns (GE Healthcare) for affinity purification. The elute protein in high-imidazole buffer (20 mM HEPES pH 8, 20 mM KCl, 250 mM NaCl and 500 mM imidazole) was subjected to a buffer exchange with no imidazole buffer (20 mM HEPES pH 7.5, 20 mM KCl, 200 mM NaCl) using Amicon Ultra-10K centrifugal filters (Milipore), added TEV protease in a protease:protein ratio (1:100 OD_280_) and incubated in a rotation well at 4 °C overnight ( ~ 16 h). The cleaved protein was subjected to a reverse affinity chromatography to remove the hexahistidine tag fused with GB1 and the uncleaved protein. The flowthrough was collected, concentrated to 1-2 mL using Amicon Ultra-10K centrifugal filters (Milipore) and subjected to size-exclusion chromatography (SEC) using a Superdex S200 Increase 16/600 column. The peak fractions were analyzed using a standard SDS-PAGE protocol, pooled, and concentrated with Amicon Ultra-10K centrifugal filters. Protein aliquots were flash-frozen in liquid nitrogen and then stored at − 80 °C.

### Protein crystallization

Crystallization was performed by the hanging-drop method at room temperature in 2 μL drops (1:1, protein:precipitation solution). Hexahistidine tag fused with GB1-UmPit2 crystallized at 20 mg/mL within 2 weeks in in 0.1 M Bicine and 1.6 M Ammonium sulfate at pH 9. Crystals were frozen flash-frozen in liquid nitrogen using a cryo-solution (mother solution supplemented with 20% (v/v) glycerol.

### Structure analysis by X-ray crystallography

Data were collected under cryogenic conditions at the European Synchrotron Radiation Facility (ESRF) beamline ID23-2^43^. The data were integrated and scaled using XDS and merged with XSCALE version 20250714^44^. The structure of Pit2 was determined by molecular replacement using Phenix version 1.20.1-4487^45^ integrated Phaser-MR and 5BMH as the search model. The structure was manually built in Coot v1.1.19^46^ and refined in Phenix version 1.20.1-4487^45^. All residues were found within the preferred and additionally allowed regions of the Ramachandran plot. Detailed data collection and refinement statistics are listed in Supplementary Data 2.

### Structure prediction and structural clustering

Protein structures and interactions were predicted by AlphaFold2^18^ v2.3 or AlphaFold 3^47^ v3.0.1, and visualized using PyMOL version 2.5.2 (https://pymol.org/) or ChimeraX 1.11.1^48^. FoldMason (version 4-dd3c235)^33^ was used to perform a multiple structural alignment using the top 20 hits from a Gpe1 (A0A0D1E6B3) Foldseek^32^ search against the AFDB50 database and systematically map structurally conserved residues across the receptor family.

### Identification of 7TM proteins in *U. maydis*

To systematically identify candidate seven-transmembrane (7TM) proteins in *Ustilago maydis*, we implemented a multi-step computational workflow combining topology prediction, structure modeling, and structure-based homology searches. The complete annotated proteome of *U. maydis* was first screened for transmembrane (TM) helices using TMHMM v2.0 d^49^. Proteins containing between six and eight predicted TM helices were retained for further analysis. All candidate proteins with 6–8 predicted TM helices were subjected to structure prediction using AlphaFold2^18^. For each protein, the highest-confidence model, ranked by predicted local distance difference test (pLDDT), was selected for downstream analyses. To assign putative structural homologs, each predicted structure was individually queried using Foldseek version 10-941cd33^32^. Structural similarity was evaluated based on alignment score, TM-score, and alignment coverage. Proteins were annotated according to their closest structural homologs, with particular attention to similarity to known 7TM receptor architectures. To expand the dataset and capture additional 7TM proteins that may have been missed due to inaccurate TM predictions (for example, proteins predicted to contain fewer than six or more than eight TM helices), we performed a secondary structure-based search restricted to the *U. maydis* proteome. Proteins exhibiting significant structural similarity to confirmed 7TM candidates, despite falling outside the initial TMHMM-based cutoff, were retrieved and manually inspected. Candidates demonstrating a conserved heptahelical bundle, consistent membrane orientation, and overall fold similarity were incorporated into the final 7TM dataset.

### Quantitative RT-PCR (qRT-PCR)

For qRT-PCR, cells were grown in CM media supplemented with 1% glucose to an OD_600_ = 0.5. 2 mL of this culture were supplemented with synthetic Pit2-petide (f.c. 1 µM) or DMSO as solvent control and incubated in a 15 mL falcon tube for 3.5 h on a rotary wheel analogous to the pheromone treatment procedure of *U. maydis*^50^. Cells were harvested by centrifugation and snap-frozen in liquid nitrogen, and total RNA was extracted using Trizol reagent (Invitrogen) according to the manufacturer’s instructions^31^. Residual DNA was digested with the Turbo DNA-free kit (Invitrogen), and 1 µg RNA was used for cDNA synthesis with the iScript cDNA Synthesis Kit (Bio-Rad). qRT-PCR was performed with three independent biological replicates and two technical replicates each per strain using the Takyon No ROX SYBR 2X MasterMix blue (Eurogentec) on a Bio-Rad CFX Duet Real-Time PCR system. Statistical significance was calculated using an unpaired Student’s t-test.

### Plant infection assays

*U. maydis* strains were grown in 50 mL YEPSlight medium from an overnight pre-culture to an OD_600_ of 1. Cells were harvest by centrifugation (1 671 x *g*, 5 min, room temperature), and resuspended in double-sterilized H_2_O to reach a final OD_600_ of 3. For the infection of maize plants, 250–500 μL of culture was injected into 7-day-old maize seedlings using a syringe. Disease symptoms were scored after 6- and 12-days post-infection and based on three biological replicates per *U. maydis* strain. Disease symptom scoring data are listed in Supplementary Data 3.

### GPCR activation assay in *S. cerevisiae*

The activation assays for each GPCR were performed as previously described with slight modifications^22^. The peptides from Pit2 variants were obtained through trypsin digestion (1:20, trypsin:protein ratio) 37 °C overnight ( ~ 12-15 h) in TE buffer (20 mM Tris-HCl pH 8, 2 mM EDTA) or synthesized (Genscript Biotech). Experiments were performed in 96-well microtiter plates using a microplate reader (Tecan), and OD_600_ and red fluorescence were measured after 8 h. Experiments were run in triplicate.

For dose response curves, 9 different concentrations (15 μM and 6 five-fold dilutions starting at 8 μM peptide, sterile water was used as no peptide control) of the specific synthetic peptide were used. Experiments were performed in 96-well microtiter plates using a microplate reader (Tecan), and OD_600_ and red fluorescence were measured after 20 h. A four-parameter non-linear regression model (curve fit) was used using Prism (GraphPad 5): Agonist concentration vs. response (variable slope). After analysis, EC_50_, basal activation, maximal activation and the Hill coefficient was extracted as previously described^22^.

The peptide secretion and GPCR expression strains were cultivated in SD media overnight and diluted 1:1 with sterile water. Ten microliters cells were seeded in 200 μL total volume in 96-well plates and cultured at 30 °C with 800 RPM shaking. The fluorescence intensity was measured at 20 h using a microplate reader (Tecan) with excitation at 588 nm and emission at 620 nm. The fold change was calculated by dividing OD_600_-normalized fluorescence values of Pit2 variants by that of the control (cells containing empty plasmid), with the control value set to 1.

### Reversed-phase Pit2 peptides isolation by HPLC

The mixture of tryptic-digested UmPit2 peptides were subjected to separation with a binary gradient of from 5 % to 95 % acetonitrile with a flowrate of 2 mL/min on an HPLC system (Agilent) for 60 min operated in a C-18 reverse phase column. The injection point was after 2 min and while the separation process was running, 2 mL sample was collected until fraction number 50. Fractions which contain peaks at 205 nm or 280 nm, were incubated at 98 °C with the lid open under a fume hood until the samples are dried. Dried peptides were resuspended in 100 μL PBS buffer (20 mM HEPES pH 7.4, 20 mM KCl, 100 mM NaCl and 10 mM Na_2_HPO_4_). 15 μL of each selected fraction was tested for GPCR activation assay in *S. cerevisiae* as described in the GPCR activation assay in *S. cerevisiae* section.

### Fluorescence microscopy

Laser-based epifluorescence microscopy was performed on a Zeiss Axio Observer.Z1 equipped with Prime BSI Express SCMOS camera (Visitron Systems, Puchheim, Germany). Excitation was provided by a VS-LMS4 Laser-Merge-System (Visitron Systems, Puchheim, Germany) that combines solid-state lasers targeting GFP (488 nm at 50 or 100 mW) and RFP (561 nm at 50 or 150 mW). An emission filter for GFP of 520 nm and for RFP of 631 nm was used. Image acquisition was performed using VisiView v7.0 (Visitron Systems, Puchheim, Germany).

Cells were grown in 10 ml cultures with 1 µM WWFGFTGSL peptide to an OD_600_ of 0.4 prior to fluorescence imaging. All staining was imaged using RFP excitation. Congo Red staining involved labeling 2 µl of cells with 3 mM Congo Red directly on the slide. FM4-64 staining required incubating 1 ml cell samples with 8 µM FM4-64 at room temperature over 15 min, followed by one wash with 1x PBS (137 mM NaCl, 2.7 mM KCl, 10 mM Na_2_HPO_4,_ 1.8 mM KH_2_PO_4_). TMRE staining involved incubating 1.5 ml samples with 10 µM TMRE at 28 °C over 20 minutes, followed by two washes with 1x PBS. Image processing was carried out using Fiji ImageJ v20250808-2217^51^.

### Microfluidics microscopy

Pictures were taken with an openFrame Microscopy System (Cairn GmbH, Ettlingen, Germany) equipped with the SCMOS sensor KINTEIX-22MM-M-C (Teledyne Photometrics, Tucson, AZ, USA) and an LDI-7 solid-state laser (89north, Williston, VT, USA) as illuminator. GFP imaging utilized 470 nm excitation and a 510 nm emission filter. Image acquisition was performed using Micro-manager 2.0^52^. Signal visualization was enhanced by applying the 16 colors lookup table to the GFP signal. Image processing was carried out using Fiji ImageJ^51^.

Cells were grown in 10 ml cultures to an OD_600_ of 0.2. Following dilution with H2O to an OD_600_ of 0.05, 200 µl of these cells was added to a µ-Slide I^0.2^ Luer Uncoated (Ibidi, Mertinsried, Germany). NM medium containing 1 µM WWFGFTGSL peptide was continuously flushed over the cells throughout the entire duration using an EasyFlow Pump System (Fluigent, Paris, France) at a flow rate of 0.4 µl/min. Images were captured every 20 minutes over a 10-hour period.

### Microscopy data evaluation and single cell tracking

Single fungal cells in microfluidic devices were tracked with the FIEST algorithm, which uses custom-trained cellpose models and generative frame interpolation for single cell tracking. Our cellpose (v4.0.7) model was trained on 23 semi-automatically labeled images of *U. maydis* and *S. cerevisiae* cells as input ( ~ 50 cells/per image). For tracking, micrographs were converted to three-layered (R, G, B) 8-bit depth PNGs before frame interpolation or single-layered 16-bit TIFs before segmentation using the Python (version 3.10.0) package YeastVision v0.1.67. Tracking was performed on hybrid real-interpolated time series, which were downsampled to the original frame rate and used for calculating cell cycle duration, defined as the interval between two budding events. The first detection of a visible bud defined the time point of budding. Budding cells that entered the microfluidics device were excluded from the analysis. Differences between treatments were evaluated assuming each microfluidic device as a biological replicate and using the Kolmogorov-Smirnov test ${kstest}2()$, with significance at *p* < 0.05.

### Coevolution analysis

A dataset of Gpe1 homologs was obtained through an HMMER v3.4 search^53^ using the sequence of *U. maydis* Gpe1 as bait. Corresponding sequences for all identified Gpe1 homologs were retrieved via the UniProt ID mapping tool (https://www.uniprot.org/id-mapping). These sequences were compiled into a multi-FASTA file and aligned using the MUSCLE v5.3 alignment tool (https://www.ebi.ac.uk/jdispatcher/msa/muscle?stype=protein). The resulting ‘.clw’ alignment file was used as input for IQ-TREE v3.0.1 (https://iqtree.github.io/), which was run locally in a Linux environment. IQ-TREE v3.0.1 was executed with default settings, allowing automatic model selection for the dataset prior to calculating the final phylogenetic tree (see original logfile). The resulting ‘.treefile’ output was visualized in the iTOL v7.2.1 (https://itol.embl.de/) web application, and the resulting tree was exported as an ‘.svg’ file for figure preparation.

To assess the presence of Pit2 homologs, genomic regions containing Gpe1 homologs were manually examined using Ensembl Fungi (https://fungi.ensembl.org/). When a Pit2 homolog was identified, the corresponding gene names and protein sequences were listed (Supplementary Data 6) and used for further alignments using MUSCLE v5.3 to identify sequence conservation in the consensus motif.

## Supplementary information

Supplementary Information

Supplementary Data 1-8

Supplementary Movie 1, Supplementary Movie 2

Description of Additional Supplementary Files

Transparent Peer Review file

## Source data

Source Data

## Data Availability

Coordinates and structure factors have been deposited within the protein data bank (PDB) under accession codes: 9RXK. Source data are provided in this paper.

## Code availability

Original Python (version 3.10.0) and MATLAB (version v25.2) code used for image analysis and single cell tracking are available in the GitHub^54^ website under the URL: https://github.com/MirandaLab/Peptide-GPCR_system_Single_Cell_quantifications.
