# Supplementary material for: A co-evolved peptide-GPCR system senses host entry to drive fungal infection
Source: Nat Commun. 2026 Oct 9;17:10652. doi: 10.1038/s41467-026-78428-6 (PMC13652807; doi:10.1038/s41467-026-78428-6)
Supplement: Supplementary file 1 [file 41467_2026_78428_MOESM1_ESM.pdf]

Supplementary Information for

A co-evolved peptide-GPCR system senses host entry to drive fungal infection

Gabriel Mendoza-Rojas[1], Philip Nakonz[1], Min Lu[2], Nora Marie Kühne[3], Johannes Postma[1], Naomi Shtakser[1], Max Heinen[1], Manav Patel[4], Orlando Arguello-Miranda[4], Kai Heimel[3], Sonja Billerbeck[2,5] and Florian Altegoer[1*]

Affiliations:

[1] Heinrich-Heine University Düsseldorf, Institute of Microbiology, Cluster of Excellence on Plant Sciences, Düsseldorf, Germany

[2] University of Groningen, Faculty of Science and Engineering, Groningen, Netherlands

[3] Georg-August University Göttingen, Microbial Cell Biology, Göttingen, Germany

[4] NC State University, Plant and Microbial Biology, Raleigh, USA

[5] Imperial College London, Department of Bioengineering, London, United Kingdom

*Correspondence to: altegoer@hhu.de

The PDF file includes:

Supplementary figure 1 to 8

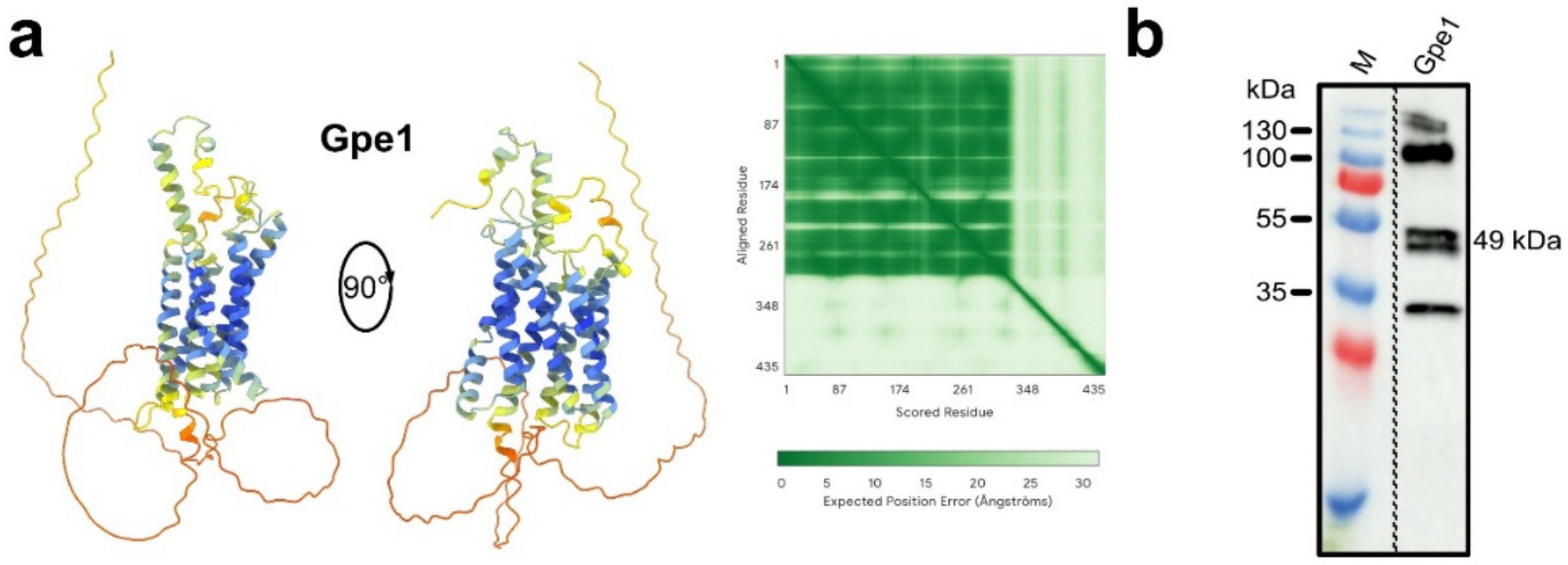

Supplementary Figure 1. Expression and structure prediction of Gpe1 a. Structure prediction of Gpe1 (A0A0D1E6B3). Shown is the model colored by predicted local distance difference test (pLDDT). The predicted aligned error (PAE) matrix is shown on the right. **b.** Western blot of Gpe1 expression in *S. cerevisiae.* M, protein marker. The experiment was repeated independently three times with similar results.

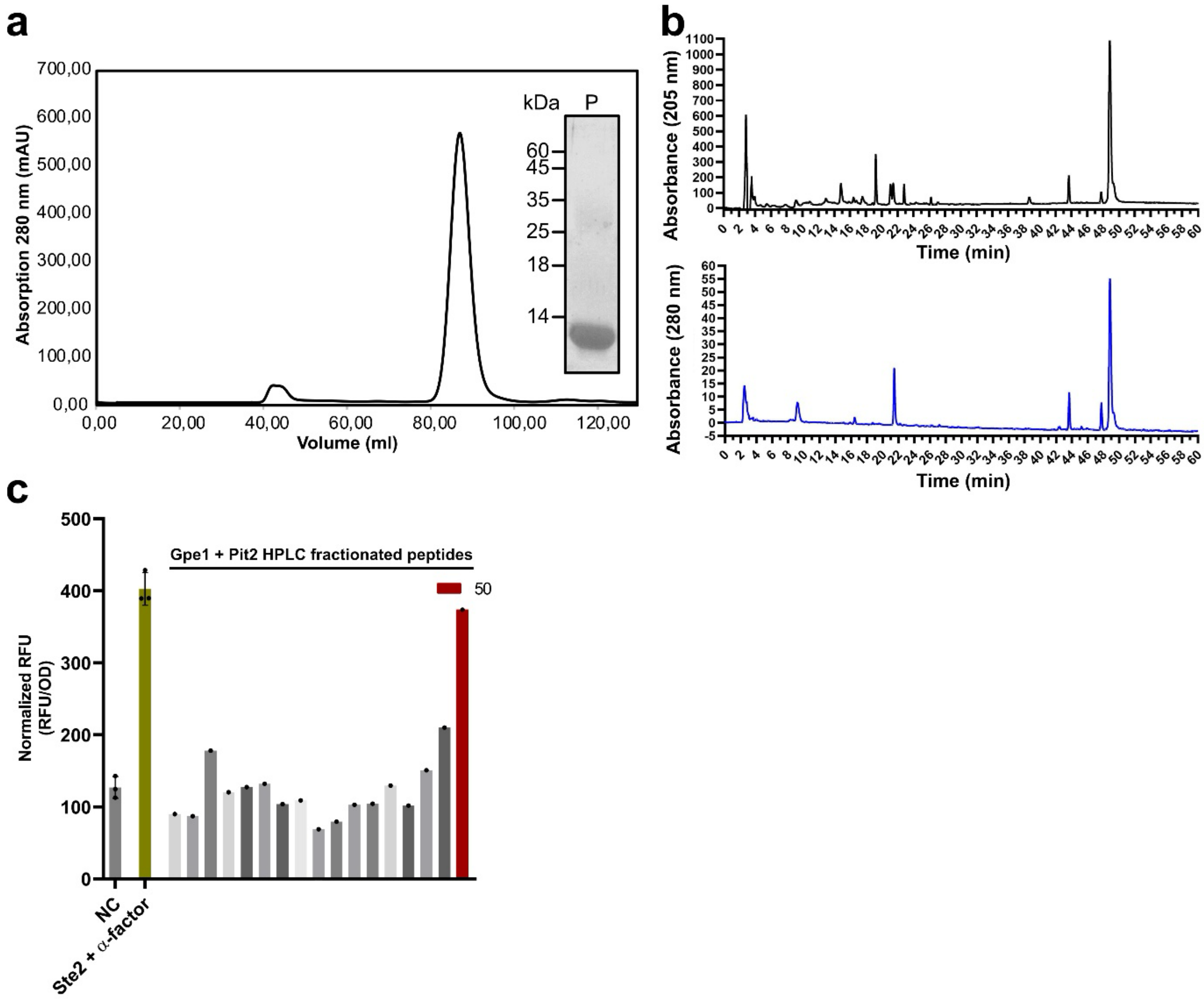

Supplementary Figure 2. Identification of Gpe1-activating Pit2 peptides. a. Size-exclusion chromatography (SEC) profile of purified Pit2 protein, showing a single monodisperse peak. Inset: Coomassie-stained SDS–PAGE gel of the peak fraction (P). **b.** HPLC analysis of Pit2-derived peptides monitored at 205 nm (top) and 280 nm (bottom), demonstrating the presence of distinct peptide species. **c.** Functional assay comparing Gpe1 receptor activation in response to fractionated peptides. RFU values are normalized to cell density (OD). Gpe1 only responds to fraction 50. Ste2 activation by the α-factor (green bar), serves as control. NC, Negative control, background strain of the yeast biosensor system. Normalized RFU values are presented as mean of three independent biological experiments with SD as error bars, except for the peptide-fractionation samples, which were analyzed once. The results obtained with these fractions were subsequently confirmed using synthetic peptide Pit2.5. Source data from the fluorescence-based yeast reporter assays are provided as a Source Data file.

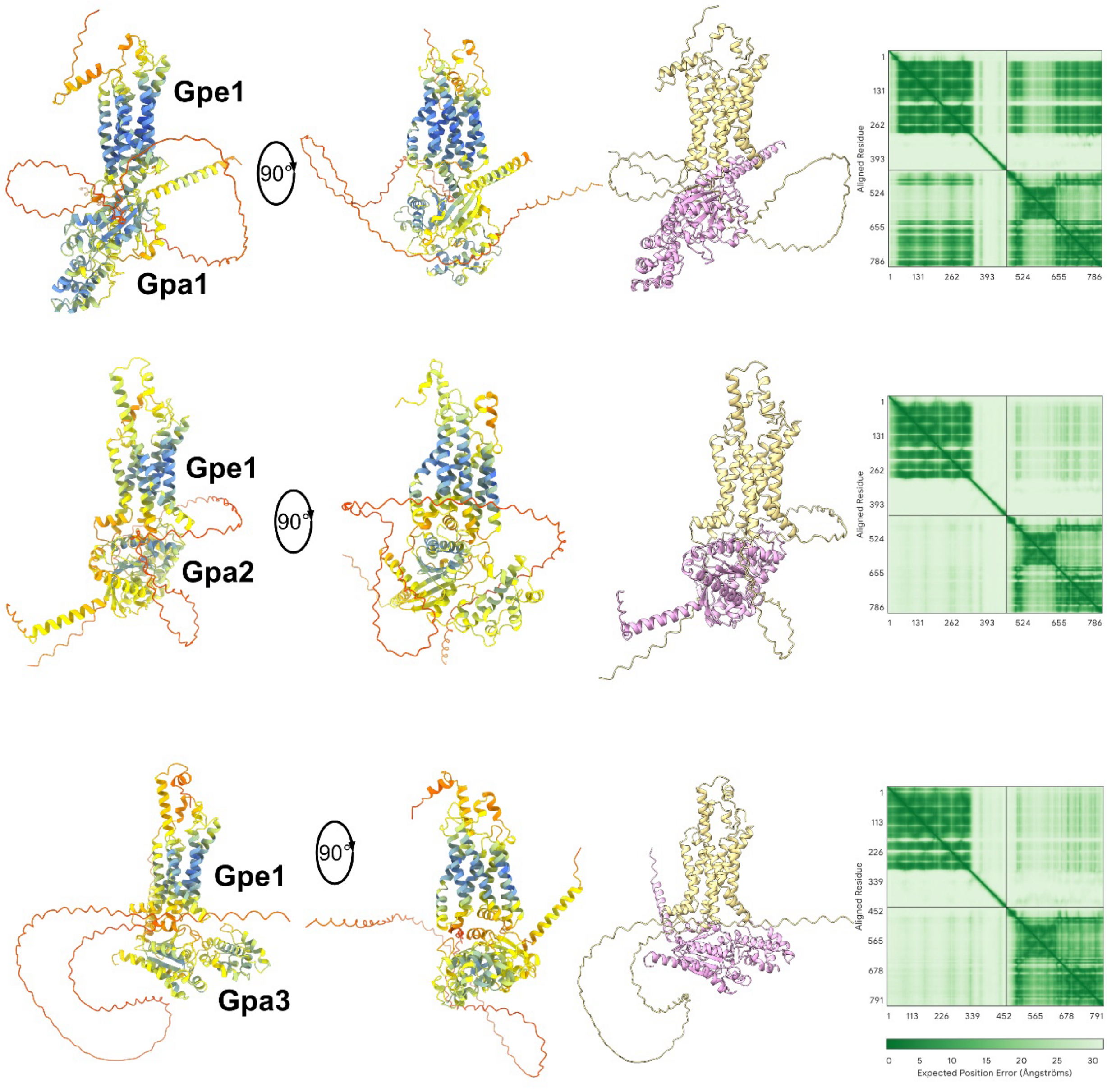

Supplementary Figure 3. Structure predictions of Gpe1 alone and in complex with Gpa1, Gpa2 and Gpa3 from *U. maydis*. Models are colored either by predicted local distance difference test (pLDDT) score or by molecule, with Gpe1 shown in yellow and the Gα subunits in pink. The corresponding predicted aligned error (PAE) matrices are shown on the right.

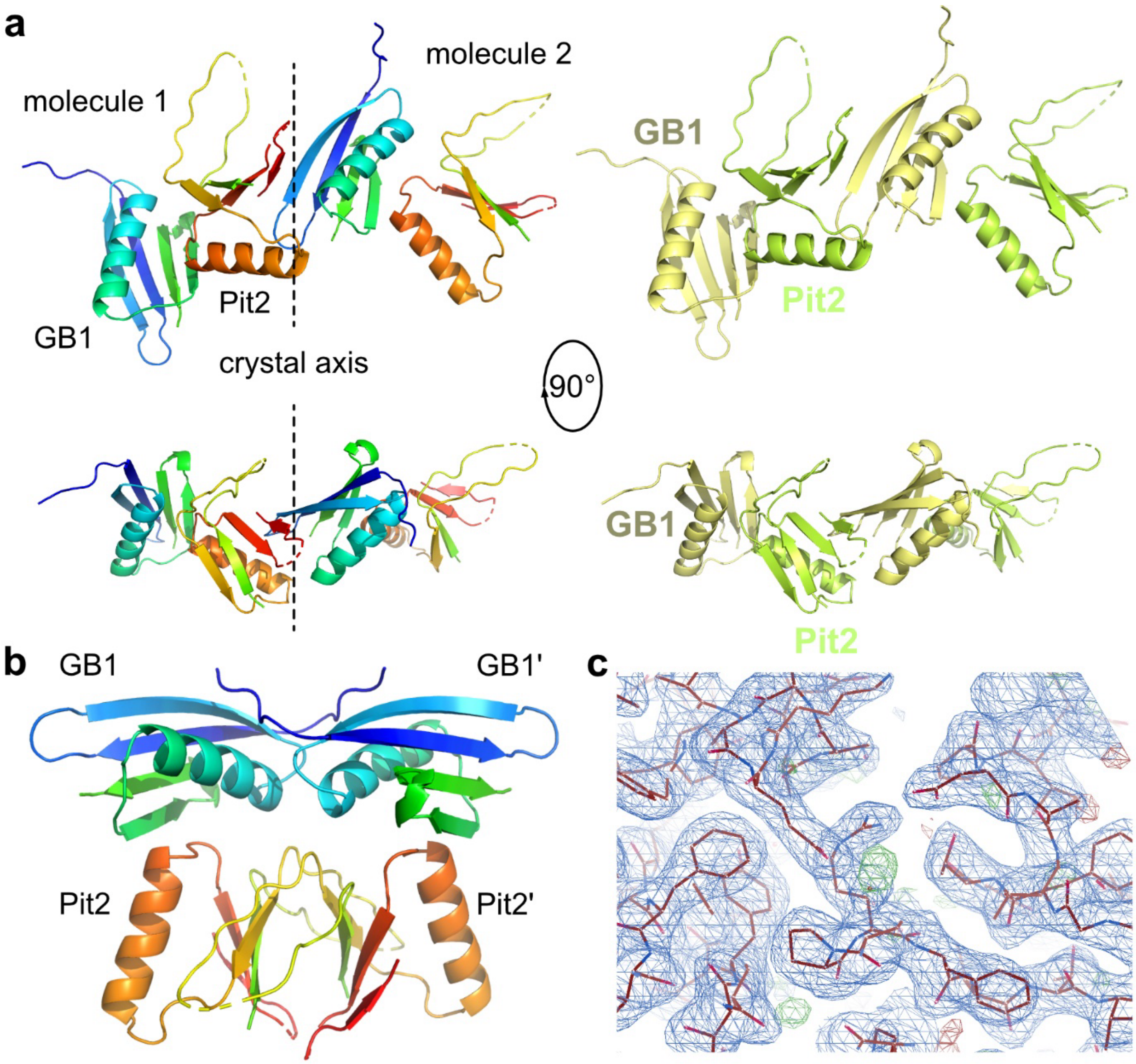

Supplementary Figure 4. Crystallographic analysis of GB1-UmPit2. a. Display of crystallographic symmetry mates of fusion construct of Pit2 with the B1 domain (GB1), GB1-Pit2, showing that the β-strand 2 of a neighboring GB1 molecule performs a β-completion with β5 of Pit2 stabilizing crystal contacts. **b.** Homodimer of GB1-Pit2 displayed in cartoon style and colored in rainbow from N- to C-terminus. **c.** Representative region of the $2F_{obs} - F_{calc}$ electron-density map for the GB1–Pit2 crystal structure, contoured at 0.99 σ and shown in blue. Positive and negative $2F_{obs} - F_{calc}$ difference density is shown in green and red, respectively, contoured at 3.0 σ. The refined atomic model is shown as sticks.

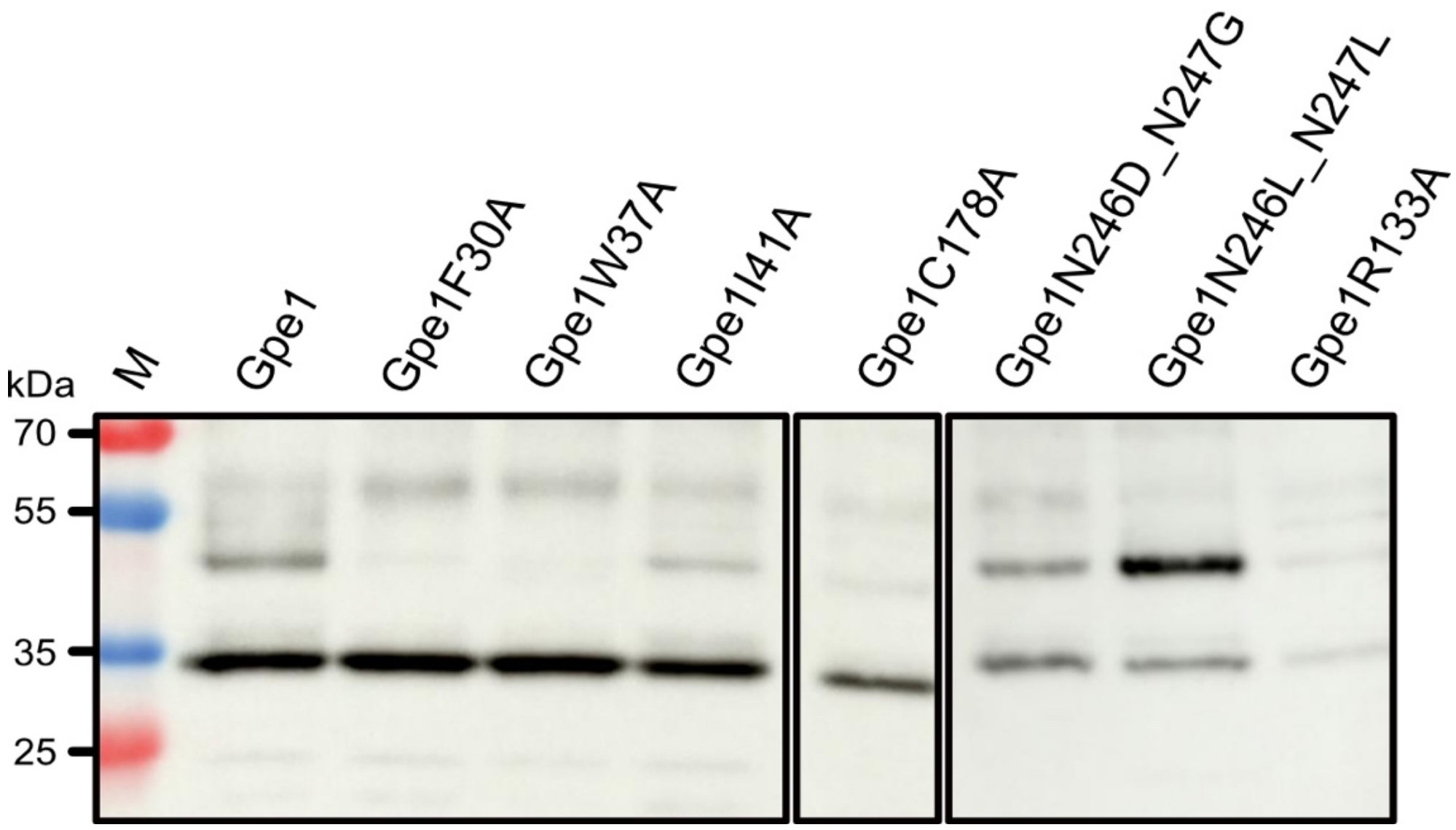

Supplementary Figure 5. Western blot of Gpe1 variant expression in *S. cerevisiae*. M, protein marker. The experiment was repeated independently three times with similar results.

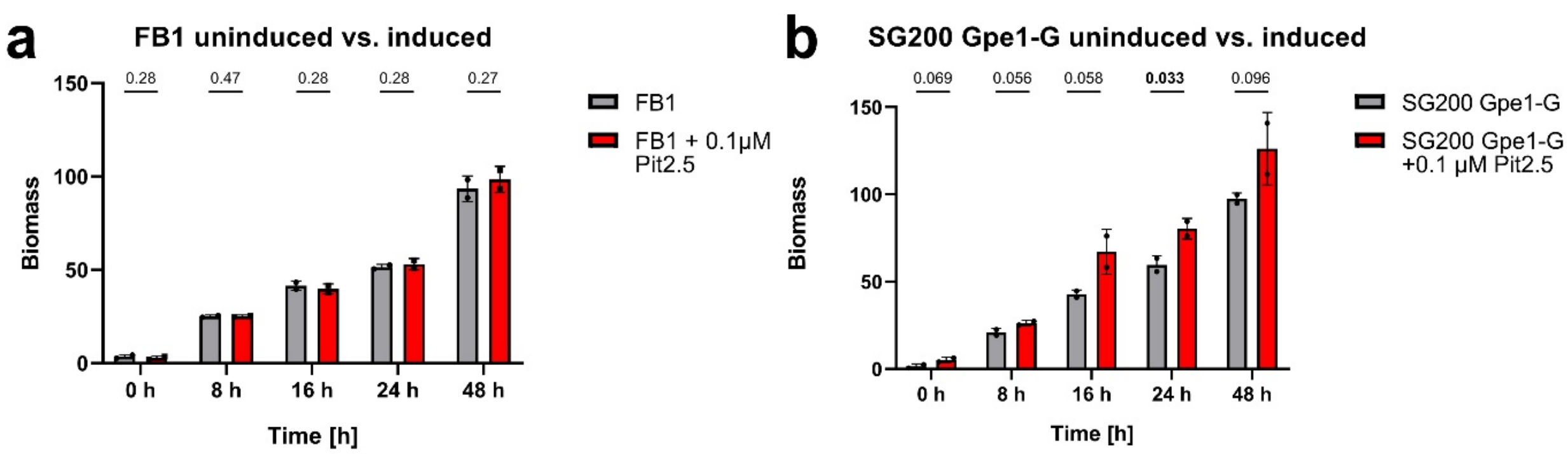

Supplementary Figure 6. Stimulation of Gpe1 by Pit2 increases fungal biomass. a. *U. maydis* FB1 grown in absence (grey) and presence (red) of 0.1 µM WWFGFTGSL (Pit2.5) peptide. No significant changes between the two conditions are observed. **b.** *U. maydis* SG00 Gpe1-G grown in absence (grey) and presence (red) of 0.1 µM WWFGFTGSL (Pit2.5) peptide. Peptide stimulation increases fungal biomass with a significant (p < 0.05) change after 24h. Error bars are represented with SD of two biological replicates. P-values obtained from two tailed paired student t-test statistical analysis are depicted above each column.

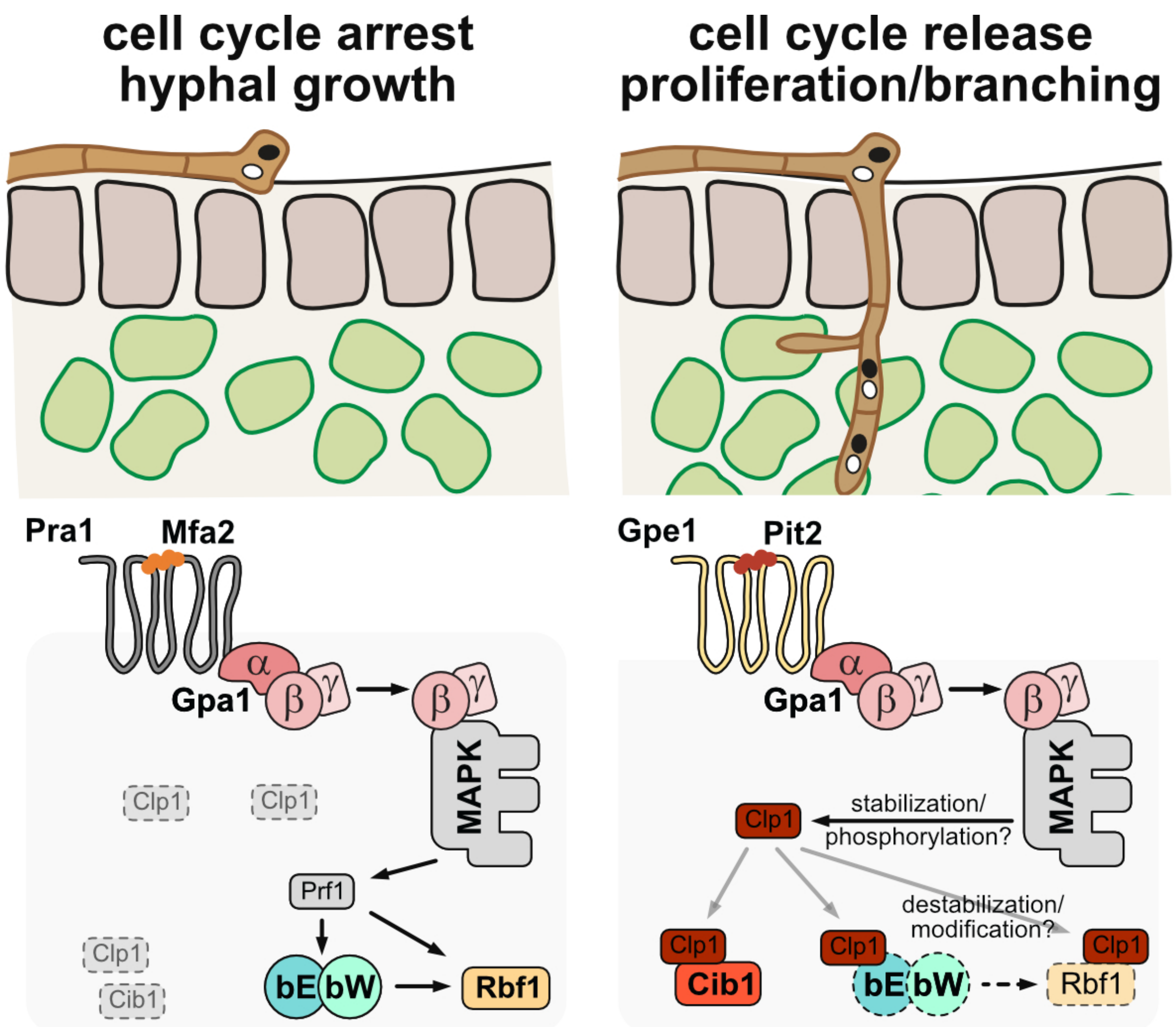

Supplementary Figure 7. Schematic model of the Gpe1/Pit2 function. Dashed boxes indicate destabilization/modification of the respective proteins.

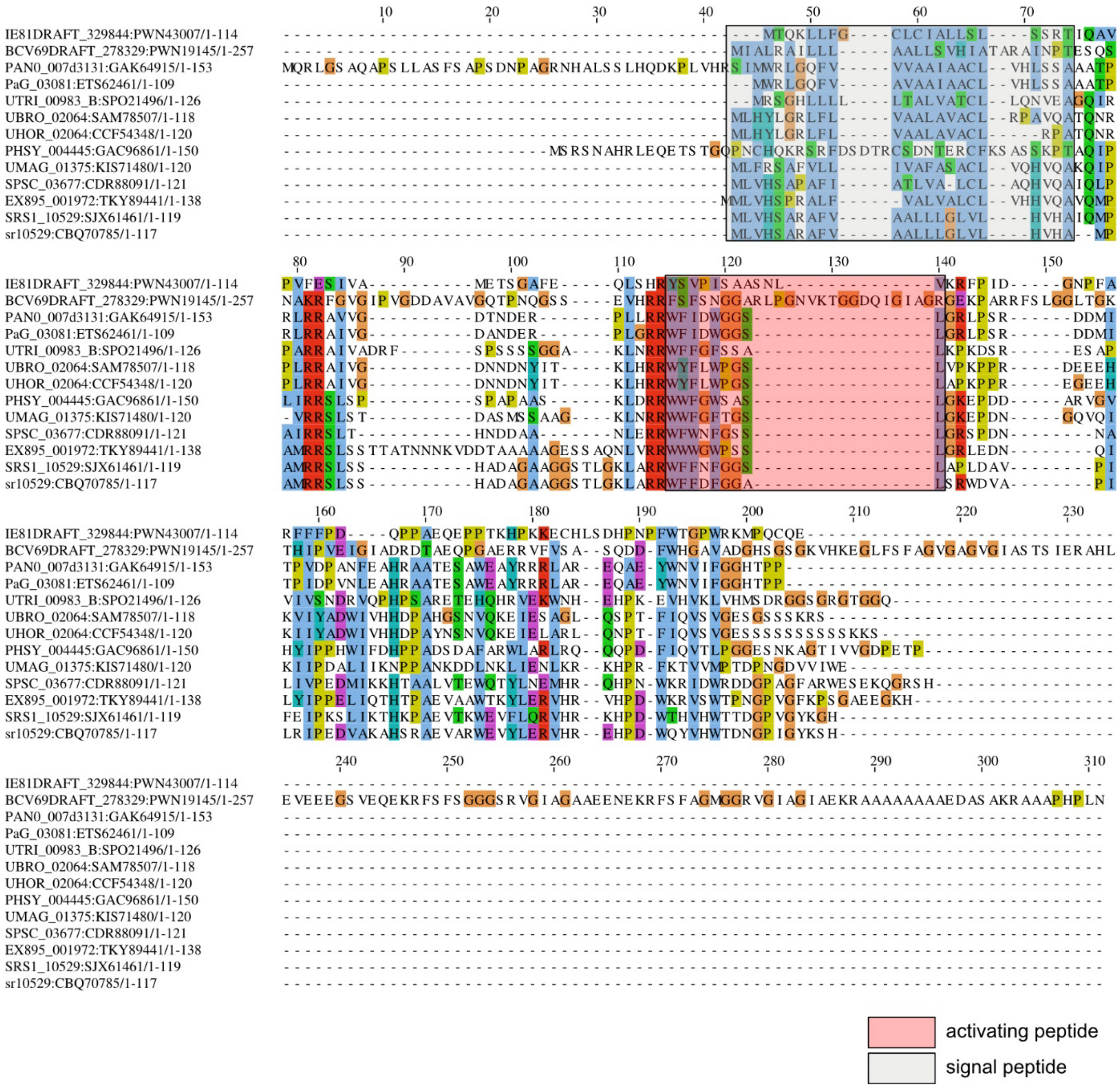

Supplementary Figure 8. Sequence alignment of Pit2 homologs. Amino acid sequence alignment of Pit2 colored according to the CLUSTAL coloring scheme. The regions containing the signal peptide and the activating Gpe1 ligand are framed in grey and red boxes, respectively.
