# Supplementary material for: A co-evolved peptide-GPCR system senses host entry to drive fungal infection
Source: Nat Commun. 2026 Oct 9;17:10652. doi: 10.1038/s41467-026-78428-6 (PMC13652807; doi:10.1038/s41467-026-78428-6)
Supplement: Supplementary file 2 [file 41467_2026_78428_MOESM4_ESM.pdf]

Description of Additional Supplementary Files:

Supplementary Data 1: List of 7TM proteins identified in *Ustilago maydis*

Supplementary Data 2: Crystallographic data collection and refinement statistics of Pit2

Supplementary Data 3: Plant infection disease scoring

Supplementary Data 4: Time required to complete a cell division in each treatment per field of view (FOV). Fungal cells divided significantly faster upon Pit2 peptide exposure as judged by the time between two budding events compared across n = 3 using a Kolmogorov–Smirnov (KS) test, each biological replicate contained at least 24 cells.

Supplementary Data 5: Relative expression of mating-type and unfolded protein response marker genes following Pit2 peptide treatment. Normalized relative expression values are provided for the indicated genes in SG200 and Gpe1–GFP-expressing *Ustilago maydis* cells cultured in the absence (−) or presence (+) of the Pit2-derived peptide. Three biological replicates. eIF2b was used for normalization of gene expression.

Supplementary Data 6: Distribution of Gpe1 and associated Pit2 homologs across basidiomycete fungi. Gpe1 homologs identified by sequence-based searches are listed together with their UniProt accessions, entry names, gene identifiers, and source organisms. Where an associated Pit2 homolog was identified, its gene identifier and protein sequence are provided.

Supplementary Data 7: Foldseek results from the structure-based search for Gpe1 homologs. Complete Foldseek output from the search of the predicted Gpe1 structure against the AlphaFold Protein Structure Database clustered at 50% sequence identity (AFDB50).

Supplementary Data 8: Plasmids, oligonucleotides, and microbial strains used in this study.

Supplementary Movie 1: Representative Pit2 peptide-exposed single cell U. Maydis time series in microfluidics. Top right: phase contrast channel with mask contour overlaid in yellow on top of the tracked cell. Top left: GFP fluorescence channel displaying the expression of Gpe1-GFP. Bottom left plot: single cell size quantification as the area in pixels occupied by the tracked cell. Bottom right, single cell mean fluorescence quantification as the 12 average intensity of

the pixels occupied by the tracked cell in the GFP channel. Notice the cell divides four times vs three times in the control (Video 2)

Supplementary Movie 2: Representative control single cell U. Maydis time series not exposed to Pi2 peptides in microfluidics. Top right: phase contrast channel with mask contour overlaid in yellow on top of the tracked cell. Top left: GFP fluorescence channel displaying the expression of Gpe1-GFP. Bottom left plot: single cell size quantification as the area in pixels occupied by the tracked cell. Bottom right, single cell mean fluorescence quantification as the average intensity of the pixels occupied by the tracked cell in the GFP channel. Notice the cell divides three times vs four times in the Pit2-exposed strain (Video 1).
