# Supplementary material for: A co-evolved peptide-GPCR system senses host entry to drive fungal infection
Source: Nat Commun. 2026 Oct 9;17:10652. doi: 10.1038/s41467-026-78428-6 (PMC13652807; doi:10.1038/s41467-026-78428-6)
Supplement: Supplementary file 3 [file 41467_2026_78428_MOESM5_ESM.pdf]

nature portfolio

Peer Review File

A co-evolved peptide-GPCR system senses host entry to drive fungal infection

Corresponding Author: Dr Florian Altegoer

This file contains all reviewer reports in order by version, followed by all author rebuttals in order by version.

Version 0:

Reviewer comments:

Reviewer #1

(Remarks to the Author)
This study identifies a co-evolved GPCR signaling mechanism in Ustilago maydis in which host protease–released peptides derived from the fungal effector Pit2 activate the GPCR Gpe1, enabling the fungus to sense host entry and promote proliferation during infection. Overall, this is an interesting study supported by a lot of experiments and analyses. The identification of a host-gated peptide-GPCR signaling mechanism represents an important advance in our understanding of fungal host sensing. While major conclusions are supported by the data, several mechanistic aspects, particularly regarding downstream signaling and the in-planta validation of host protease dependency, are not yet fully resolved and would benefit from additional clarification or more cautious interpretation. I have the following specific comments for the authors to address.

1 While the study provides compelling evidence that Pit2-derived peptides activate the GPCR Gpe1, the downstream signaling pathway remains largely inferred. The proposed involvement of Gpa1, MAPK signaling, and Clp1 is mainly based on structural predictions and phenotypic observations. More direct biochemical evidence (e.g., Gα activation, MAPK phosphorylation, or Clp1 function) would strengthen the conclusions.

2 A major conclusion of the study is that Gpe1 activation is strictly host-dependent and gated by host papain-like cysteine protease (PLCP) activity. However, direct in planta evidence linking host protease activity to Gpe1 signaling remains limited. Additional experiments or clearer discussion addressing how host PLCP activity contributes to Gpe1 activation during infection would strengthen this key aspect of the model.

3 Pit2 is proposed to act both as a PLCP inhibitor and as a precursor of the Gpe1-activating peptide. However, the relative contribution of these two activities to fungal virulence is not fully disentangled.

4 The interaction between Gpe1 and the Gα subunit (Gpa1) is inferred solely from structural modeling and lacks direct experimental validation. Supporting evidence from co-immunoprecipitation, yeast two-hybrid assays, or genetic epistasis analyses would greatly strengthen the conclusion that Gpe1 signals through Gpa1. In the absence of such data, this interaction should be more explicitly presented as a prediction.

5 The predominant ~110 kDa band observed in Western blot analyses is interpreted as evidence for Gpe1 homodimerization. However, clarification is needed regarding whether this conclusion is supported by the experimental conditions used (e.g., native versus reducing SDS-PAGE). Given that GPCRs often exhibit atypical migration behavior, alternative explanations cannot be excluded. Additional validation, or a more cautious interpretation, would be appropriate. Moreover, since a putative dimerization interface is proposed based on structural modeling, testing whether mutations in key interface residues disrupt dimer formation and yield a monomeric species would further support this claim.

6 Although the data convincingly show that Gpe1 activation accelerates cell cycle progression, the proposed mechanistic link to the Clp1/Cib1 regulatory module appears inferential. Direct evidence demonstrating that Pit2-Gpe1 activation affects Clp1 stability, activity, or downstream Cib1-dependent transcription is currently lacking. Experiments assessing Clp1 protein levels, post-translational modification, or expression of Cib1 target genes following Pit2 stimulation would substantially strengthen this proposed signaling hierarchy. Alternatively, a more cautious interpretation in the Discussion would help delineate this pathway.

7 It remains unclear whether Gpe1 signaling is mainly routed through the Clp1/Cib1 regulatory module, or whether Gpe1 activation leads to a broader range of downstream transcriptional or signaling responses, with Clp1/Cib1 being only one outcome highlighted in this study.

8 Gpe1 homologs are broadly conserved among smut fungi, whereas Pit2 appears restricted to species closely related to U. maydis. This raises the question of whether Pit2 represents the sole ligand for Gpe1 in U. maydis, or whether additional, as yet unidentified ligands may exist, particularly in species lacking Pit2 homologs. In this context, it would be helpful to discuss how the similar virulence defects observed in Δgpe1 and Δpit2 strains can be reconciled with the possibility of alternative ligands.

Reviewer #2

(Remarks to the Author)
Menodoza-Rojas et al describe a novel receptor (that is proposed to be a GPCR) involved in a system for detection of suitable growth conditions for the plant pathogenic fungus Ustilago maydis that causes corn smut. U. maydis secretes the protein Pit2, which is cleaved by plant proteases. A specific Pit2 peptide released binds to the proposed GPCR Gpe1, causing fungal proliferation. This is a novel system that allows the fungus to sense it's environment and ensures it is ideal for proliferation.

The authors provide several lines of experimental and computational evidence to support the proposed mechanism. An AlphaFold model of Gpe1 based on its amino sequence predicted an integral membrane protein with 7 transmembrane alpha-helices that has architectural similarities to the alpha-factor mating receptor Sterile 2 (Ste2), a GPCR from Saccharomyces cerevisiae (the only fungal GPCR for which a structure is known). Alphafold also predicted that the U. maydis G protein Gpa1 coupled to Gpe1 and that this was apparently more likely than coupling of the other G proteins Gpa2 and Gpa3. A peptide that caused activation of Gpe1 in a S. cerevisiae reporter system was identified by peptide fraction of Pit2-digested peptides and mass spectrometry. Synthetic peptides were then used to define the exact sequence that gave the best activity (WWFGFTGSL). This peptide is buried in the structure of Pit2 as determined by X-ray crystallography. Mutation of residues in the proposed orthosteric binding pocket of Gpe1 affected signalling by Gpe1 with a maximum 10-fold decrease in EC50 and 6-fold reduction in Emax. This was corroborated by looking at symptoms on maize leaves upon infection by U. maydis containing the C178A mutation, a pit2 deletion and a gpe1 deletion. Further data show that Gpe1 activation by the peptide WWFGFTGSL causes receptor internalisation (a characteristic feature of many GPCRs) and promotes proliferation of U. maydis. Phylogenetic data suggest that the Gpe1-Pit2 system is widespread in smut fungi.

The manuscript is very clearly written with good supporting figures. I also found the Gpe1-Pit2 system both incredibly exciting and interesting, as there are potential ramifications well beyond corn smut fungi. However, as a GPCR structural biologist I find there is too much acceptance of the Alphafold model of Gpe1 by the authors that needs to be toned down in further iterations of the manuscript.

Major points

1. Alphafold is an amazing tool, but it relies heavily on published structures where co-ordinates have been deposited in the PDB. The more structures published similar to the subject (in this case Gpe1), the better the predictions. Remember that Alphafold does not incorporate any biophysics into its predictions, it is looking for patterns just like ChatGPT. Thus predictions of some class A GPCRs like the beta-adrenoceptors, adenosine and serotonin receptors, which are very similar and have 100s of structures, are very good with RMSDs of about 0.5-1 Å compared to the actual structure. In contrast, the fungal receptors (Class D) are represented by only a single structure (Ste2) and this is different to any of the mammalian GPCR structures from Classes A, B, C and F. The other problem is that the diversity of GPCR structures in fungi could be even greater than that observed in mammalian systems. One phylogenetic analysis divided fungal receptor into 10 different groups, whereas another divided it into 17. The combination of these factors means that the prediction of Gpe1 is probably not very good and would need substantial supporting data from biophysical studies (beyond the scope of the current work) to convince me that it is reasonable. The RMSD between the AlphaFold model and a Ste2 monomer is 6 Å, which indicates that no specific details from Ste2 can be used to infer biology of Gpe1. For this paper, I therefore suggest the following:
• Discuss the limitations of the AlphaFold model; the seven transmembrane helix architecture is likely to be correct but any further conclusions are far too speculative to be included
• Perform a structural search using for example the Dali Server to search for structural homologues to the model, rather than assuming Ste2 is the closest homologue
• Delete the dimer models of Gpe1; TM1 of the Gpe1 model bears little similarity to that of Ste2 (or any other GPCR structure for that matter)
• Delete the models coupled to G proteins and remove the conclusion that it couples to Gpa1 (further experimental evidence is required for this). The reason I do not believe the models is that the binding mode of Gpa1 to Gpe1 has never been observed in GPCR-G protein complexes; the C-terminus of the G protein that is the major specificity determinant needs to be totally engaged by the receptor, which is not the case in the model.

2. The Alphafold model probably does not have the accuracy to provide an accurate template for ligand docking. In addition, predicting the binding mode of peptides in GPCR structures is incredibly difficult to achieve. These two observations makes it unlikely that the binding mode of the peptide depicted is correct. Two facts support this conclusion. Firstly, if as the authors suggest Gpe1 is similar to Ste2, then the binding mode of the peptide is completely different to the binding of the agonist alpha-factor to Ste2. In the Gpe1-model, the N-terminal portions of the peptide are in the centre of the helical bundle,

whereas in Ste2 the N-terminus of Alpha-factor is at the periphery which is key to the activation of the receptor. The mutagenesis results support the binding mode of the peptide in the Gpe1 model, but these are extremely limited and could be interpreted in a number of different ways, considering that an EC50 is being measured (reporting on ligand binding, receptor activation and downstream signalling) compared to e.g. radioligand binding (reporting on solely ligand binding). The binding mode is supported by the data in Fig.1e far more than is discussed in the manuscript. Firstly, any additional residues at the N-terminus of the peptide abolishes signalling (Pit2.1 and 2.2), which is entirely consistent with the N-terminus being buried in the centre of the receptor. Secondly residues can be added to the C-terminus of the peptide (2.3 and 2.4), which reduces binding, but does not abolish it; this is consistent with the orientation of the C-terminus of the peptide that extends towards the entrance to the predicted orthosteric binding pocket. The statistical significance of these comparisons will need to be determined. So for the new version of the manuscript I would suggest:
• Giving a more nuanced discussion of the predicted mode of peptide binding to the Gpe1 model
• Use existing data to support the proposed binding mode
• Generate new data looking at the binding mode of peptides based on Pit2.5 with a small extension at its N-terminus (e.g. a single alanine residue) which should not bind and changing the Trp and Phe residues in the peptide to alanine which should increase EC50 and decrease Emax significantly. Increasing the size of the Gly, Ser and Thr residues with e.g. Leu would also be expected to have similar effects.
3. The oligomeric state of membrane proteins is incredibly difficult to determine from using only data from an SDS-PAGE gel. Heating membrane proteins in an SDS-PAGE loading buffer (even at 50˚C) can result in full denaturation and aggregation, resulting in decreased mobility on the gel so that the protein looks like a dimer. There are very few membrane protein oligomers that remain oligomeric in the presence of SDS. Ste2 has the largest interface between dimers for any GPCR where the interface is formed of transmembrane alpha-helices. It includes the GxxxG motif first observed in glycophorin A. Ste2 runs as a monomer on SDS-PAGE when the samples are left in loading buffer at room temperature for 15 minutes. Some of the glycophorin A runs as a dimer, and some as a monomer. Gpe1 does not have GxxxG motif. At the other end of the spectrum, AmtB is a remarkably stable trimer that can only be dissociated by boiling the sample in SDS. I suggest the following:
• Have four samples of Gpe1 in SDS-PAGE buffer and leave them for either 15 minutes at 20˚C, 37˚C or 50˚C, with the final sample being boiled for 2 minutes. Compare the results on SDS-PAGE.
Hopefully this will clarify the matter, but this is still only an indication. If the dimer is as stable as the authors claim, then it should be straightforward to purify it in a mild detergent and show by SEC-MALS that it is indeed a dimer.

Minor points

1. Lines 54-56. The methodology for the structure based genome wide screen needs to be put into the methods
2. Lines 57-60. What is the UMAG number of Gpe1 (reference to table)
3. Lines 72-73. Briefly describe the approach here for the receptor activation assay.
4. Line 85. Explain the Ste2 control i.e. alpha factor is a full agonist.

Version 1:

Reviewer comments:

Reviewer #1

(Remarks to the Author)
My previous comments have been addressed, and I have no further suggestions.

Reviewer #2

(Remarks to the Author)
The authors have made many alterations to the manuscript and have included some new data that has strengthened the conclusions drawn in the manuscript. There are just a number of minor comments that need to be addressed to improve clarity and precision of the text.

1. Line 76: change to '…also predicted to be connected to TM3…'
2. Lines 81-83. At this point explain that Gpe1 can couple to Gpa1 from S. cerevisiae and that this leads to the induction of red fluorescent protein expression (move lines 95-97 and clarify)
3. Line 81: change to '…BAR1, SST2…'
4. Line 86: change to '…phenotypically indical reduction in virulence…'
5. Line 88: change to '…and we therefore purified…'
6. Lines 97-99: the structure predictions do not support the observation given that the engagement of the alpha5 helix of the G protein does not bind in a manner similar to any other GPCR-G protein complex (as mentioned in my first review). The authors should either remove this or do the experiments to confirm this.
7. Line 241: change to …a structure based homology search based on the Alphafold model of Gpe1 using Foldseek and restricted to fungi.'
8. Line 243: I do not understand 'carried a DUF6533' Please clarify.
9. Line 245: replace 'high-confidence' with 'some'. (High-confidence is an RMSD of < 1Å)
10. Line 725: change to 'A potential disulfide bond…'

11. For all graphs where there are error bars, please state in the legend the number of biological replicates, the number of technical replicates and the type of error depicted.
12. Fig. 1e: The red portion defining WWFGFTGSL is absent from the lefthand panel and in the righthand panel the red area also includes sequence downstream from the peptide. Please correct.
13. Fig 2d: The data for Pit2.5 (10A) cannot be used as no saturation is observed due to the absence of the two points at high concentrations of peptide. Also, please give the starting amino acid residue in '10A'.
14. Fig. 4e. The open symbols for the N246L+N247L and R133A are too faint; please improve the colour intensity. What does ND mean? If no binding was observed, it is probably better to use NB (no binding) rather than ND, which in English normally stands for Not Determined.
15. Fig 4e and Fig 2d: For the mutants showing lower activity, it is important to show that the cell surface expression of the mutants is similar to wild type. Please include the data
16. Extended Data Fig. 2c: The Ste2 bar is green, not red. Only fraction 50 is shown, not 46 and 50 as stated in the legend.

Open Access This Peer Review File is licensed under a Creative Commons Attribution 4.0 International License, which permits use, sharing, adaptation, distribution and reproduction in any medium or format, as long as you give appropriate credit to the original author(s) and the source, provide a link to the Creative Commons license, and indicate if changes were made.
In cases where reviewers are anonymous, credit should be given to 'Anonymous Referee' and the source.
The images or other third party material in this Peer Review File are included in the article's Creative Commons license, unless indicated otherwise in a credit line to the material. If material is not included in the article's Creative Commons license and your intended use is not permitted by statutory regulation or exceeds the permitted use, you will need to obtain permission directly from the copyright holder.
To view a copy of this license, visit https://creativecommons.org/licenses/by/4.0/

REVIEWER COMMENTS

Reviewer #1 (Remarks to the Author):

This study identifies a co-evolved GPCR signaling mechanism in Ustilago maydis in which host protease–released peptides derived from the fungal effector Pit2 activate the GPCR Gpe1, enabling the fungus to sense host entry and promote proliferation during infection. Overall, this is an interesting study supported by a lot of experiments and analyses. The identification of a host-gated peptide-GPCR signaling mechanism represents an important advance in our understanding of fungal host sensing. While major conclusions are supported by the data, several mechanistic aspects, particularly regarding downstream signaling and the in-planta validation of host protease dependency, are not yet fully resolved and would benefit from additional clarification or more cautious interpretation. I have the following specific comments for the authors to address.

1 While the study provides compelling evidence that Pit2-derived peptides activate the GPCR Gpe1, the downstream signaling pathway remains largely inferred. The proposed involvement of Gpa1, MAPK signaling, and Clp1 is mainly based on structural predictions and phenotypic observations. More direct biochemical evidence (e.g., Gα activation, MAPK phosphorylation, or Clp1 function) would strengthen the conclusions.

We thank this reviewer for pointing towards this weak spot in our manuscript. We have performed additional experiments that more clearly establish the link towards Clp1. A detailed answer is provided below.

2 A major conclusion of the study is that Gpe1 activation is strictly host-dependent and gated by host papain-like cysteine protease (PLCP) activity. However, direct in planta evidence linking host protease activity to Gpe1 signaling remains limited. Additional experiments or clearer discussion addressing how host PLCP activity contributes to Gpe1 activation during infection would strengthen this key aspect of the model.
3 Pit2 is proposed to act both as a PLCP inhibitor and as a precursor of the Gpe1-activating peptide. However, the relative contribution of these two activities to fungal virulence is not fully disentangled.

This is clearly an important question and I agree that we have not fully addressed this in the previous version of our manuscript. At present, we cannot fully separate these two functions in planta. However, several lines of evidence suggest that Gpe1 activation represents a major virulence-relevant output of Pit2 processing: In the respective single deletion strains of gpe1 and pit2, expression of the respective other gene is not affected, which was already reported by Doehlemann et al. (2011). Thus, the strong virulence defect of the gpe1 deletion strain cannot be explained by a loss of pit2 expression. In this strain, Pit2 is still present at wild-type levels, yet infection is almost abolished. Similarly, several Gpe1 point mutants generated in this study strongly reduce receptor activation but should not interfere with the PLCP-inhibitory function of Pit2. These mutants also show strongly impaired infection phenotypes. Together, these observations argue that loss of Gpe1 signaling has a major impact on virulence even when Pit2-mediated PLCP inhibition should remain intact.

We therefore conclude that Gpe1 activation is likely a major function of Pit2 during infection, whereas the relative contribution of PLCP inhibition remains less clearly resolved. Importantly, these activities are not mutually exclusive. Misas-Villamil et al. (2019) reported the presence of the peptide that we identify here as Gpe1-activating after incubation of Pit2 with PLCP-containing lysates (Sup. Figure 5). This suggests that PLCP-mediated Pit2 processing can generate both the previously described PLCP-inhibitory peptide and the Gpe1-activating peptide. We have revised the manuscript to better reflect this dual-function model and to explicitly state that future experiments will be required to genetically or biochemically uncouple PLCP inhibition from Gpe1 activation in planta.

4 The interaction between Gpe1 and the Gα subunit (Gpa1) is inferred solely from structural modeling and lacks direct experimental validation. Supporting evidence from co-immunoprecipitation, yeast two-hybrid assays, or genetic epistasis analyses would greatly strengthen the conclusion that Gpe1 signals through Gpa1. In the absence of such data, this interaction should be more explicitly presented as a prediction.

We completely agree that we overstated a potential coupling of Gpe1 and Gpa1, which was only based on alphafold predictions. However, Gpe1 couples to ScGpa1 (being the UmGpa1 homolog) without any modifications in the yeast reporter system, supporting the idea that Pit2-activated Gpe1 might couple to UmGpa1. The yeast reporter assays therefore provide direct evidence of an interaction between Gpe1 and Gpa1. Earlier work on the four G-alpha subunits of U. maydis (Regenfelder 1997) showed that only deletion of gpa3 showed reduced virulence, however these strains also suffer from severe morphological defects complicating epistasis analyses. Likely, signal transduction involves other components (e.g. arrestins) and simply showing a connection with one of the G-proteins is insufficient to explain the complete signal transduction pathway. We have therefore rephrased and toned down the potential Gpa1 coupling.

5 The predominant ~110 kDa band observed in Western blot analyses is interpreted as evidence for Gpe1 homodimerization. However, clarification is needed regarding whether this conclusion is supported by the experimental conditions used (e.g., native versus reducing SDS-PAGE). Given that GPCRs often exhibit atypical migration behavior, alternative explanations cannot be excluded. Additional validation, or a more cautious interpretation, would be appropriate. Moreover, since a putative dimerization interface is proposed based on

structural modeling, testing whether mutations in key interface residues disrupt dimer formation and yield a monomeric species would further support this claim.

We have removed the Gpe1 dimer as there was insufficient evidence for homodimer formation.

6 Although the data convincingly show that Gpe1 activation accelerates cell cycle progression, the proposed mechanistic link to the Clp1/Cib1 regulatory module appears inferential. Direct evidence demonstrating that Pit2-Gpe1 activation affects Clp1 stability, activity, or downstream Cib1-dependent transcription is currently lacking. Experiments assessing Clp1 protein levels, post-translational modification, or expression of Cib1 target genes following Pit2 stimulation would substantially strengthen this proposed signaling hierarchy. Alternatively, a more cautious interpretation in the Discussion would help delineate this pathway.
7 It remains unclear whether Gpe1 signaling is mainly routed through the Clp1/Cib1 regulatory module, or whether Gpe1 activation leads to a broader range of downstream transcriptional or signaling responses, with Clp1/Cib1 being only one outcome highlighted in this study.

*As outlined above, we have now performed additional experiments to understand the link between Gpe1 activation and Clp1. We now quantified transcript levels of selected mating type pathway-associated genes and UPR marker genes after Pit2 peptide treatment in SG200 WT and Gpe1-G expressing cells (**Fig.3d**). These experiments showed that constitutive Gpe1 expression reduces transcript levels of most tested mating type pathway-associated genes, with the notable exception of clp1, which is approximately five-fold increased relative to SG200 WT. Importantly, Pit2 peptide stimulation further increased clp1 expression in the Gpe1-expressing background, but not in the SG200 WT control. In contrast, UPR marker genes were not detectably affected under these conditions. These data support a specific effect of Pit2–Gpe1 activation on clp1 transcription, but they do not demonstrate activation of the full Clp1/Cib1 regulatory module. We have therefore modified the manuscript accordingly. We now state that Pit2–Gpe1 signaling may promote re-initiation of fungal proliferation at least in part by increasing clp1 transcript levels, providing a potential connection to the established Clp1/Cib1 module that controls cell-cycle re-entry after plant penetration. We also agree that Gpe1 signaling may not be exclusively routed through Clp1/Cib1 but given the complexity of this network, we believe a thorough investigation is required to address this in the future.*

8 Gpe1 homologs are broadly conserved among smut fungi, whereas Pit2 appears restricted to species closely related to U. maydis. This raises the question of whether Pit2 represents the sole ligand for Gpe1 in U. maydis, or whether additional, as yet unidentified ligands may exist, particularly in species lacking Pit2 homologs. In this context, it would be helpful to discuss how the similar virulence defects observed in Δgpe1 and Δpit2 strains can be reconciled with the possibility of alternative ligands.

That is an interesting point. Our current data support Pit2-derived peptide signaling as the major, and possibly dominant, route of Gpe1 activation in U. maydis. This is consistent with the very similar virulence defects observed for Δpit2 and Δgpe1 strains: loss of either the receptor or the Pit2-derived activating peptide strongly compromises infection. Thus, under the infection conditions tested here, any potential alternative ligand cannot compensate for the absence of Pit2 or Gpe1. We therefore interpret the phenotypic similarity of Δpit2 and Δgpe1 as evidence that Pit2-dependent activation accounts for the major virulence-relevant Gpe1 signaling input in U. maydis. This of course, does not rule out that Gpe1 activation or signaling might be influenced by additional alternative ligands.
In addition, we have now performed a broader and structure-based search for GPCRs using Gpe1, which identified a wide range of GPCR candidates across basidiomycetes. This suggests that Gpe1 belongs to a larger and previously underappreciated receptor family with similarities to mammalian rhodopsin GPCRs. Given that a few variations in Gpe1 might already completely change the ligand spectrum, we consider it likely that Gpe1 versions of smut species lacking Pit2 have evolved a completely different ligand spectrum whilst retaining the same activation mechanisms.

Reviewer #2 (Remarks to the Author):

Menodoza-Rojas et al describe a novel receptor (that is proposed to be a GPCR) involved in a system for detection of suitable growth conditions for the plant pathogenic fungus Ustilago maydis that causes corn smut. U. maydis secretes the protein Pit2, which is cleaved by plant proteases. A specific Pit2 peptide released binds to the proposed GPCR Gpe1, causing fungal proliferation. This is a novel system that allows the fungus to sense it's environment and ensures it is ideal for proliferation.

The authors provide several lines of experimental and computational evidence to support the proposed mechanism. An AlphaFold model of Gpe1 based on its amino sequence predicted an integral membrane protein with 7 transmembrane alpha-helices that has architectural similarities to the alpha-factor mating receptor Sterile 2 (Ste2), a GPCR from Saccharomyces cerevisiae (the only fungal GPCR for which a structure is known). Alphafold also predicted that the U. maydis G protein Gpa1 coupled to Gpe1 and that this was apparently more likely than coupling of the other G proteins Gpa2 and Gpa3. A peptide that caused activation of Gpe1 in a S. cerevisiae reporter system was identified by peptide fraction of Pit2-digested peptides and mass spectrometry. Synthetic peptides were then used to define the exact sequence that gave the best activity (WWFGFTGSL). This peptide is buried in the structure of Pit2 as determined by X-ray crystallography. Mutation of residues in the proposed orthosteric binding pocket of Gpe1 affected signalling by Gpe1 with a maximum 10-fold decrease in EC50 and 6-

fold reduction in Emax. This was corroborated by looking at symptoms on maize leaves upon infection by U. maydis containing the C178A mutation, a pit2 deletion and a gpe1 deletion. Further data show that Gpe1 activation by the peptide WWFGFTGSL causes receptor internalisation (a characteristic feature of many GPCRs) and promotes proliferation of U. maydis. Phylogenetic data suggest that the Gpe1-Pit2 system is widespread in smut fungi.

The manuscript is very clearly written with good supporting figures. I also found the Gpe1-Pit2 system both incredibly exciting and interesting, as there are potential ramifications well beyond corn smut fungi. However, as a GPCR structural biologist I find there is too much acceptance of the Alphafold model of Gpe1 by the authors that needs to be toned down in further iterations of the manuscript.

Major points

1. Alphafold is an amazing tool, but it relies heavily on published structures where co-ordinates have been deposited in the PDB. The more structures published similar to the subject (in this case Gpe1), the better the predictions. Remember that Alphafold does not incorporate any biophysics into its predictions, it is looking for patterns just like ChatGPT. Thus predictions of some class A GPCRs like the beta-adrenoceptors, adenosine and serotonin receptors, which are very similar and have 100s of structures, are very good with RMSDs of about 0.5-1 Å compared to the actual structure. In contrast, the fungal receptors (Class D) are represented by only a single structure (Ste2) and this is different to any of the mammalian GPCR structures from Classes A, B, C and F. The other problem is that the diversity of GPCR structures in fungi could be even greater than that observed in mammalian systems. One phylogenetic analysis divided fungal receptor into 10 different groups, whereas another divided it into 17. The combination of these factors means that the prediction of Gpe1 is probably not very good and would need substantial supporting data from biophysical studies (beyond the scope of the current work) to convince me that it is reasonable. The RMSD between the AlphaFold model and a Ste2 monomer is 6 Å, which indicates that no specific details from Ste2 can be used to infer biology of Gpe1. For this paper, I therefore suggest the following:

• Discuss the limitations of the AlphaFold model; the seven transmembrane helix architecture is likely to be correct but any further conclusions are far too speculative to be included

We thank the reviewer for this important and constructive comment. We fully agree that our previous version placed too much weight on the AlphaFold model of Gpe1 and that, in the absence of an experimentally determined structure, detailed structural conclusions have to be interpreted with substantial caution. We also agree that the large RMSD between the Gpe1 model and Ste2 indicates that specific mechanistic conclusions cannot be transferred from Ste2 to Gpe1. Our assumptions were mainly (mis)guided by the fact that both receptors have peptide ligands and act on the cell cycle but they apparently belong to completely different GPCR classes. In fact, the reviewer's comment prompted us to examine this point in more detail. Our broader structure-based searches suggest that Gpe1 belongs to a larger group of fungal GPCRs widespread across basidiomycetes.

• Perform a structural search using for example the Dali Server to search for structural homologues to the model, rather than assuming Ste2 is the closest homologue

*We thank the reviewer for this excellent suggestion. To address this point, we have now performed structure-based searches using the predicted Gpe1 model as query using Foldseek. As stated above, this analysis revealed structural homologs of Gpe1 across basidiomycete fungi. We then used FoldMason to construct a structural-sequence alignment of a subset of these potential GPCRs, which was incredibly informative. Most notably, the structural alignment uncovered (among others) a conserved R3.50 residue and a microswitch-like interaction network equivalent to core activation motifs reported for mammalian GPCRs (**Fig.4d**). This was unexpected and substantially strengthened our interpretation of Gpe1 as part of a broader fungal GPCR family with a potentially conserved activation logic. We also tested this observation experimentally and show that variations of the R3.50 or potentially interacting residues impair Gpe1 activation (**Fig.4e**). Our analysis therefore established an unexpected link to mammalian class a/rhodopsin receptors, which might be very informative to investigate the diversity of fungal GPCRs from an evolutionary perspective.*

• Delete the dimer models of Gpe1; TM1 of the Gpe1 model bears little similarity to that of Ste2 (or any other GPCR structure for that matter)

We have deleted the dimer models.

• Delete the models coupled to G proteins and remove the conclusion that it couples to Gpa1 (further experimental evidence is required for this). The reason I do not believe the models is that the binding mode of Gpa1 to Gpe1 has never been observed in GPCR-G protein complexes; the C-terminus of the G protein that is the major specificity determinant needs to be totally engaged by the receptor, which is not the case in the model.

We agree that the evidence for Gpa1-coupling solely through alphafold predictions is not sufficient. However, the Gpe1 activation in the yeast reporter strain results in solid activation of the MAPK pathway through the endogenous Gpa1, which is the homolog of U. maydis Gpa1. We have therefore relocated and toned down the respective paragraph: "Remarkably, tryptic digestion of Pit2 peptides - resulting in a similar cleavage pattern

compared to PLCP treatment 12,13 - increased the reporter signal to ~100% at tenfold lower peptide concentration (Fig.1d), demonstrating robust Gpe1 activation by processed Pit2 fragments in S. cerevisiae. Furthermore, the yeast reporter strain carries and unmodified version of ScGpa1, which is the homolog of U. maydis Gpa1 24. Reporter response therefore demonstrates a functional interaction of Gpe1 with Gpa1. Structure predictions using the U. maydis Ga-subunits further support this observation (Extended Data Fig.3)."

2. The Alphafold model probably does not have the accuracy to provide an accurate template for ligand docking. In addition, predicting the binding mode of peptides in GPCR structures is incredibly difficult to achieve. These two observations makes it unlikely that the binding mode of the peptide depicted is correct. Two facts support this conclusion. Firstly, if as the authors suggest Gpe1 is similar to Ste2, then the binding mode of the peptide is completely different to the binding of the agonist alpha-factor to Ste2. In the Gpe1-model, the N-terminal portions of the peptide are in the centre of the helical bundle, whereas in Ste2 the N-terminus of Alpha-factor is at the periphery which is key to the activation of the receptor. The mutagenesis results support the binding mode of the peptide in the Gpe1 model, but these are extremely limited and could be interpreted in a number of different ways, considering that an EC50 is being measured (reporting on ligand binding, receptor activation and downstream signalling) compared to e.g. radioligand binding (reporting on solely ligand binding). The binding mode is supported by the data in Fig.1e far more than is discussed in the manuscript. Firstly, any additional residues at the N-terminus of the peptide abolishes signalling (Pit2.1 and 2.2), which is entirely consistent with the N-terminus being buried in the centre of the receptor. Secondly residues can be added to the C-terminus of the peptide (2.3 and 2.4), which reduces binding, but does not abolish it; this is consistent with the orientation of the C-terminus of the peptide that extends towards the entrance to the predicted orthosteric binding pocket. The statistical significance of these comparisons will need to be determined. So for the new version of the manuscript I would suggest:

- Giving a more nuanced discussion of the predicted mode of peptide binding to the Gpe1 model
- Use existing data to support the proposed binding mode
- Generate new data looking at the binding mode of peptides based on Pit2.5 with a small extension at its N-terminus (e.g. a single alanine residue) which should not bind and changing the Trp and Phe residues in the peptide to alanine which should increase EC50 and decrease Emax significantly. Increasing the size of the Gly, Ser and Thr residues with e.g. Leu would also be expected to have similar effects.

*We thank this reviewer for suggesting these peptide versions and especially for pointing out that we did not make sufficient use of our data to support the potential binding mode. Importantly, we now made better use of our existing peptide truncation data and generates additional peptide variants (**Fig. 2c,**d). Addition of a single alanine to the N-terminus strongly reduced Gpe1 activation (although it did not abolish it as observed for arginine residues at the N-terminus), supporting the idea that the free N-terminal region of the peptide is critical for productive receptor engagement. Moreover, substitution of the N-terminal hydrophobic residues, including the tryptophan/phenylalanine-containing region, strongly impaired activation. In contrast, substitutions closer to the C-terminus had more moderate and differential effects. Dose-response analyses of selected variants further supported this interpretation: some substitutions changed apparent potency, whereas others primarily reduced maximal activation. We have updated the paragraph accordingly.*

3. The oligomeric state of membrane proteins is incredibly difficult to determine from using only data from an SDS-PAGE gel. Heating membrane proteins in an SDS-PAGE loading buffer (even at 50˚C) can result in full denaturation and aggregation, resulting in decreased mobility on the gel so that the protein looks like a dimer. There are very few membrane protein oligomers that remain oligomeric in the presence of SDS. Ste2 has the largest interface between dimers for any GPCR where the interface is formed of transmembrane alpha-helices. It includes the GxxxG motif first observed in glycophorin A. Ste2 runs as a monomer on SDS-PAGE when the samples are left in loading buffer at room temperature for 15 minutes. Some of the glycophorin A runs as a dimer, and some as a monomer. Gpe1 does not have GxxxG motif. At the other end of the spectrum, AmtB is a remarkably stable trimer that can only be dissociated by boiling the sample in SDS. I suggest the following:

- Have four samples of Gpe1 in SDS-PAGE buffer and leave them for either 15 minutes at 20˚C, 37˚C or 50˚C, with the final sample being boiled for 2 minutes. Compare the results on SDS-PAGE.

Hopefully this will clarify the matter, but this is still only an indication. If the dimer is as stable as the authors claim, then it should be straightforward to purify it in a mild detergent and show by SEC-MALS that it is indeed a dimer.

These are very helpful suggestions. However, we do not see sufficient evidence supporting oligomerization of Gpe1 and accordingly removed this from the manuscript.

Minor points

1. Lines 54-56. The methodology for the structure based genome wide screen needs to be put into the methods

We apologize for not having included this methodology. It now appears in the section: Identification of 7TM proteins in U. maydis.

2. Lines 57-60. What is the UMAG number of Gpe1 (reference to table)

The UMAG identifier was added (UMAG_01374).

3. Lines 72-73. Briefly describe the approach here for the receptor activation assay.

We have added the following section: "To enable ligand identification, Gpe1 was expressed in an engineered Saccharomyces cerevisiae strain to test receptor activation through a fluorescent reporter [17]. This strain is devoid of both endogenous mating GPCR genes (STE2 and STE3), all pheromone genes (MFA1/2 and MFALPHA1/MFALPHA2), BAR1 and SST2 and FAR1 [17]. An inducible red fluorescent protein (yEmRFP) under control of the FUS1 promoter serves as readout".

4. Line 85. Explain the Ste2 control i.e. alpha factor is a full agonist.

We have added this information.

REVIEWER COMMENTS

Reviewer #2 (Remarks to the Author):

Major points

1. Line 76: change to '...also predicted to be connected to TM3...'

The sentence has been changed accordingly

2. Lines 81-83. At this point explain that Gpe1 can couple to Gpa1 from S. cerevisiae and that this leads to the induction of red fluorescent protein expression (move lines 95-97 and clarify)

We thank the reviewer for pointing out that the reporter system required further explanation at this stage of the manuscript. We have moved the relevant information to this paragraph and clarified the assay principle.

3. Line 81: change to '...BAR1, SST2...'

The punctuation has been corrected.

4. Line 86: change to '...phenotypically indical reduction in virulence...'

The sentence has been corrected.

5. Line 88: change to '...and we therefore purified...'

The sentence has been changed accordingly.

6. Lines 97-99: the structure predictions do not support the observation given that the engagement of the alpha5 helix of the G protein does not bind in a manner similar to any other GPCR-G protein complex (as mentioned in my first review). The authors should either remove this or do the experiments to confirm this.

We thank the reviewer for reiterating this important concern. We agree that the predicted complex cannot establish Gpe1–Gpa1 coupling or its precise interaction geometry. Nevertheless, we note that shallow α5-helix engagement is not unprecedented among experimentally determined GPCR–G-protein complexes (see comparison below). In the fungal Ste2–Gpa1 complex, the α5 helix binds to a ledge formed by the intracellular ends of TM3–TM5 rather than entering the deep cytoplasmic cleft characteristic of many class A receptors. Peripheral or shallow engagement has also been observed in $GABA_B$–Gi complexes. These structures demonstrate that deviation from the canonical class A geometry does not, by itself, exclude productive G-protein engagement, although they clearly do not validate the predicted Gpe1–Gpa1 complex.
Although Gpe1 contains conserved features suggestive of evolutionary relationships to class A GPCRs, its conformational dynamics and mode of G-protein engagement may differ substantially from those of canonical mammalian class A receptors. We consider AlphaFold-based complex predictions valuable hypothesis-generating tools, particularly for understudied fungal GPCRs for which little structural information is currently available. Retaining this analysis may therefore help prioritize Gpa1 for future functional and structural studies. In response to the reviewer's concern, we have revised the text to make explicit that the prediction identifies only a candidate coupling partner and requires experimental validation:

"Comparative structure predictions with the U. maydis Gα subunits suggest UmGpa1 as a candidate interaction partner for Gpe1; however, this hypothesis and the predicted interaction geometry require experimental validation (Supplementary Fig. 3)."

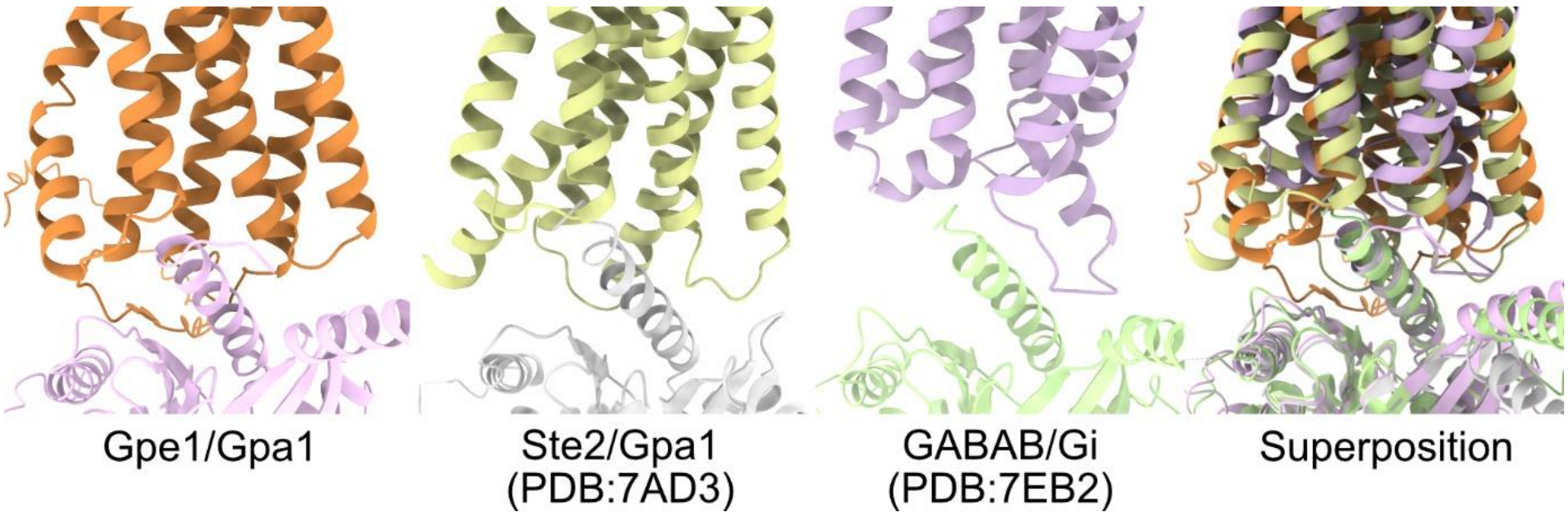

7. Line 241: change to ...a structure based homology search based on the Alphafold model of Gpe1 using Foldseek and restricted to fungi.'

The sentence has been changed accordingly.

8. Line 243: I do not understand 'carried a DUF6533' Please clarify.

The sentence has been revised to make clear that these uncharacterized proteins have a pfam domain of unknown function: 'Many of these proteins are characterized by a domain of unknown function (DUF6533)…'

9. Line 245: replace 'high-confidence' with 'some'. (High-confidence is an RMSD of < 1Å)

The term "high-confidence structural similarity" has been replaced with "some structural similarity,"

10. Line 725: change to 'A potential disulfide bond...'

The sentence has been changed accordingly.

11. For all graphs where there are error bars, please state in the legend the number of biological replicates, the number of technical replicates and the type of error depicted.

We apologize for not having included this graph details and experiment information. We have revised the figure legends for all quantitative graphs to specify the number of independent biological replicates where applicable, and whether the error bars represent standard deviation or standard error of the mean.

12. Fig. 1e: The red portion defining WWFGFTGSL is absent from the lefthand panel and in the righthand panel the red area also includes sequence downstream from the peptide. Please correct.

We have corrected both panels so that the WWFGFTGSL sequence is shown in red in the schematic representation and only the residues corresponding to this sequence are colored red in each protomer of the Pit2 structure.

13. Fig 2d: The data for Pit2.5 (10A) cannot be used as no saturation is observed due to the absence of the two points at high concentrations of peptide. Also, please give the starting amino acid residue in '10A'.

We agree that a reliable EC50 value cannot be determined for this variant because the response does not reach a clear upper plateau. Higher peptide concentrations impaired the growth of the yeast reporter strain and were therefore excluded from the analysis.

14. Fig. 4e. The open symbols for the N246L+N247L and R133A are too faint; please improve the colour intensity. What does ND mean? If no binding was observed, it is probably better to use NB (no binding) rather than ND, which in English normally stands for Not Determined.

We have increased the intensity and visibility of the open symbols representing the N246L/N247L and R133A variants. We also agree that "ND" is ambiguous. We have therefore replaced "NB" with "no binding"

15. Fig 4e and Fig 2d: For the mutants showing lower activity, it is important to show that the cell surface expression of the mutants is similar to wild type. Please include the data

We agree that receptor expression and trafficking are important considerations when interpreting the activities of the Gpe1 variants. We therefore performed membrane-fraction Western blot analyses in several independent experiments. Mostly, variants could be detected in yeast membrane preparations, supporting their expression. However, signal intensities varied considerably between biological replicates. This likely reflects, at least in part, the variable expression associated with the 2µ-based plasmid system, but also extraction-based variations. Importantly, Western blot signal intensity did not consistently correlate with activity in the reporter assays. Moreover, detection in a total membrane fraction cannot distinguish plasma-membrane-localized receptor from protein retained in intracellular membranes. We therefore do not consider these experiments sufficient to demonstrate quantitatively equivalent surface localization of every variant. We have added the available membrane-expression controls as Supplementary Fig. 5 and now explicitly acknowledge this limitation: 'However, reduced activity of individual variants may also reflect effects on receptor stability or intracellular trafficking competence.'
Accordingly, loss of reporter activity is interpreted as showing that the respective residue is required for receptor function in the yeast assay, but we cannot exclude altered receptor stability or trafficking as a contributing factor. Addressing this conclusively would require establishing a dedicated surface-localization assay, for example extracellular epitope labeling combined with flow cytometry. However, establishing these techniques will require a considerable amount of time and effort and therefore exceed the scope of the current manuscript.

16. Extended Data Fig. 2c: The Ste2 bar is green, not red. Only fraction 50 is shown, not 46 and 50 as stated in the legend

The figure legend has been corrected to state that the Ste2 control is shown in green. The legend has also been revised to clarify that only fraction 50 is shown.
